# Cryptosporidiosis threat under climate change in China: prediction and validation of habitat suitability and outbreak risk for human-derived *Cryptosporidium* based on ecological niche models

**DOI:** 10.1186/s40249-023-01085-0

**Published:** 2023-04-11

**Authors:** Xu Wang, Yanyan Jiang, Weiping Wu, Xiaozhou He, Zhenghuan Wang, Yayi Guan, Ning Xu, Qilu Chen, Yujuan Shen, Jianping Cao

**Affiliations:** 1 National Institute of Parasitic Diseases, Chinese Center for Disease Control and Prevention (Chinese Center for Tropical Diseases Research); Key Laboratory of Parasite and Vector Biology, National Health Commission of the People’s Republic of China; World Health Organization Collaborating Center for Tropical Diseases, Shanghai, 200025 China; 2 National Institute for Viral Disease Control and Prevention, Chinese Center for Disease Control and Prevention, Beijing, 102206 China; 3 School of Life Sciences, East China Normal University, Shanghai, 200241 China; 4 Key Laboratory of Public Health Safety, Fudan University, Ministry of Education, Fudan University Center for Tropical Disease Research, Fudan University School of Public Health, Shanghai, 200031 China; 5 The School of Global Health, Chinese Center for Tropical Diseases Research, Shanghai Jiao Tong University School of Medicine, Shanghai, 200025 China

**Keywords:** *Cryptosporidium*, Cryptosporidiosis, Ecological niche models, Climate change, One Health, Maxent

## Abstract

**Background:**

Cryptosporidiosis is a zoonotic intestinal infectious disease caused by *Cryptosporidium* spp., and its transmission is highly influenced by climate factors. In the present study, the potential spatial distribution of *Cryptosporidium* in China was predicted based on ecological niche models for cryptosporidiosis epidemic risk warning and prevention and control.

**Methods:**

The applicability of existing *Cryptosporidium* presence points in ENM analysis was investigated based on data from monitoring sites in 2011–2019. *Cryptosporidium* occurrence data for China and neighboring countries were extracted and used to construct the ENMs, namely Maxent, Bioclim, Domain, and Garp. Models were evaluated based on Receiver Operating Characteristic curve, Kappa, and True Skill Statistic coefficients. The best model was constructed using *Cryptosporidium* data and climate variables during 1986‒2010, and used to analyze the effects of climate factors on *Cryptosporidium* distribution. The climate variables for the period 2011‒2100 were projected to the simulation results to predict the ecological adaptability and potential distribution of *Cryptosporidium* in future in China.

**Results:**

The Maxent model (AUC = 0.95, maximum Kappa = 0.91, maximum TSS = 1.00) fit better than the other three models and was thus considered the best ENM for predicting *Cryptosporidium* habitat suitability. The major suitable habitats for human-derived *Cryptosporidium* in China were located in some high-population density areas, especially in the middle and lower reaches of the Yangtze River, the lower reaches of the Yellow River, and the Huai and the Pearl River Basins (cloglog value of habitat suitability > 0.9). Under future climate change, non-suitable habitats for *Cryptosporidium* will shrink, while highly suitable habitats will expand significantly (*χ*^*2*^ = 76.641, *P* < 0.01; *χ*^*2*^ = 86.836, *P* < 0.01), and the main changes will likely be concentrated in the northeastern, southwestern, and northwestern regions.

**Conclusions:**

The Maxent model is applicable in prediction of *Cryptosporidium* habitat suitability and can achieve excellent simulation results. These results suggest a current high risk of transmission and significant pressure for cryptosporidiosis prevention and control in China. Against a future climate change background, *Cryptosporidium* may gain more suitable habitats within China. Constructing a national surveillance network could facilitate further elucidation of the epidemiological trends and transmission patterns of cryptosporidiosis, and mitigate the associated epidemic and outbreak risks.

**Graphical Abstract:**

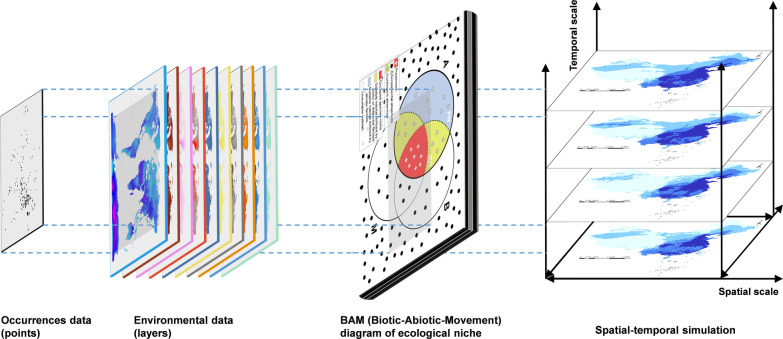

**Supplementary Information:**

The online version contains supplementary material available at 10.1186/s40249-023-01085-0.

## Background

Cryptosporidiosis is a global zoonotic parasitic disease caused by protozoan infections of *Cryptosporidium* spp*.* of the phylum Apicomplexa. *Cryptosporidium* can infect humans directly or indirectly through the fecal–oral route [1]. After entering the host, oocysts normally parasitize intestinal epithelial cells, and can cause acute gastroenteritis, abdominal pain, and diarrhea in humans [2]. Because of its highly infectious and transmissible nature, in addition to high resistance to disinfectants, cryptosporidiosis is an acute infectious disease of major concern to the international community [3]. Because cryptosporidiosis prevalence is influenced by public health conditions, it is often significantly more prevalent in low-income countries and regions, and constitutes an emerging poverty-induced parasitic disease [4]. In China, the first indigenous case of cryptosporidiosis was reported by Han et al. in 1987 [5]. As of 2018, *Cryptosporidium* infection has been reported in most provincial administrative regions in China [6]. Therefore, the disease has continuously attracted the attention of government departments and public health institutions [7]. However, due to the lack of a routine surveillance system for cryptosporidiosis, the epidemic status of cryptosporidiosis in China remains unclear [8]. Therefore, spatial model predictions based on existing data can provide theoretical support for further prevention and control programs.

Due to the lack of systematic national epidemiological surveys on cryptosporidiosis, the existence of *Cryptosporidium* in certain regions can only be determined based on existing data; however, there is no way of demonstrating that *Cryptosporidium* has never occurred in a region. For such “presence-only” data, ecological niche models (ENMs) may be the most suitable types of spatial simulation methods [9]. ENMs rely on available biogeographic information to project the environmental conditions (i.e., ecological niches) that allow organisms to survive and reproduce, and ultimately project such conditions onto the actual landscape to simulate suitable habitats [10]. Common correlational models include the Bioclimate (Bioclim) model, in which the ecological niche is defined as a hyper-rectangular range defined by the extreme values of each environmental variable [11]; a model based on genetic algorithms for rule-set production (Garp), which primarily utilizes species distribution and environmental data to run sets that produce different rules to determine the ecological needs of species and predict their potential distribution in space [12]; the Domain model, which is based on the Gower algorithm, uses a point-to-point similarity matrix to calculate the suitability of environmental variables at the target point, and obtains a discontinuous distribution interval by setting the threshold value [13]; and the maximum entropy (Maxent) model, which is based on the second law of thermodynamics, introduces the concept of information entropy, and fits the probability distribution with the maximum entropy value as the predicted species distribution [14]. Today, the application of ENMs is no longer limited to ecological studies of plants and animals, and epidemiological studies increasingly attempt to adopt and enrich ENM methods [15].

Compared to spatial epidemiology, ENMs in addition to being able to predict disease transmission risk, can determine landscape factors of disease prevalence and predict pathogen distribution patterns under environmental change scenarios [16]. This is particularly true for parasites and parasitic diseases. For example, various protozoa and helminths, such as *Toxoplasma gondii*, *Giardia lamblia*, *Trypanosoma cruzi*, *Fasciola* spp., *Anisakis* spp., and their respective hosts, have been analyzed using ENMs with satisfactory results [17–20]. Meanwhile, researchers have applied ENMs to predict the risk of common parasitic diseases such as malaria, trypanosomiasis, schistosomiasis, onchocerciasis, and filariasis [21, 22]. Therefore, ENMs have played a significant role in parasite control planning [23]. Notably, there is reportedly a significant correlation between *Cryptosporidium* bio-adaptation and climate, mainly reflected in the influence of temperature and precipitation (humidity) on activity, survival time, and dispersal dynamics of oocysts after excretion by the host and directly exposed to the environment [24–26]. For example, in areas with mild climate, the density of *Cryptosporidium* oocysts and the incidence of cryptosporidiosis may be positively correlated with temperature; specifically, the temperature may be the highest monthly temperature, the average temperature in the wet season, or even the average temperature in the previous 1–3 months. In other regions (such as North America), the study found that the survival rate and infectivity of oocysts in the environment were negatively correlated with temperature, because higher temperatures increased the mortality of *Cryptosporidium* oocysts [27, 28]. In Australia, there is a correlation between temperature and cryptosporidiosis incidence in rural areas, but not in urban areas. Furthermore, precipitation may also be correlated to *Cryptosporidium*; in equatorial and subtropical regions, the concentration of *Cryptosporidium* oocysts and the incidence rate of cryptosporidiosis are positively correlated with rainfall. Extreme weather can increase the concentration of oocysts, and heavy rainfall may cause the outbreak of cryptosporidiosis. In temperate regions, precipitation may dilute the concentration of cryptosporidium oocysts through surface water flows, resulting in a negative correlation between incidence rate and rainfall [27, 28]. Jagai et al. reported that in humid tropical climates, precipitation is a key driver of seasonal cryptosporidiosis, and the incidence of cryptosporidiosis in temperate climates peaks at high temperatures [29]. This biological characteristic of *Cryptosporidium* provides for a chance to use ENMs to predict its spatial distribution under a climate change background.

Actually, many zoonotic diseases caused by parasites are vulnerable to changes in climate, partially because of direct exposure to climate conditions in their free-living stages [30]. Climate change may be key factors affecting parasite survival, leading to parasite co-extinction, redistribution, or emergence of new infectious diseases [31]. Within some regions, parasite abundance may even increase by an order of magnitude, and some studies have successfully traced parasite invasion from tropical regions to temperate ecosystems during climate change, with native species replaced during climate change, with unpredictable ecological consequences [32]. Therefore, it is considered one of the most pressing theoretical problems in parasitic epidemiology to understand the dynamics of prevalence patterns and the distribution of parasites under climate change [33]. In the present study, we focus on *Cryptosporidium* survival trends in the context of climate change, specifically including how applicable are ENMs; how do climatic factors play a role; and what are the spatial distributions in past, present, and future periods in ecological adaptation analysis of *Cryptosporidium*? This is a notable attempt to apply ENMs in simulating the potential spatial and temporal distributions of *Cryptosporidium*, an important parasite with a relatively weak research foundation in China.

## Methods

### Extraction of bioclimatic variables

Nineteen common bioclimatic variables have been summarized based on the Assessment Report of the Intergovernmental Panel on Climate Change (IPCC), which are mainly deduced from temperature and precipitation data and can reflect the environmental constraints and their impacts on organism distribution and survival [34]. Such climate data can be obtained from the WorldClim database (https://www.worldclim.org/) [35] and downloaded from the Climatologies at the High Resolution for the Earth’s Land Surface Areas (CHELSA) database (https://chelsa-climate.org/) [43]. In the present study, the latest versions 2.1 of the two databases were used to develop the model. For the future climate data projections in both databases, a more conservative scenario, SSP126, and a more effective simulation model, MRI-ESM2-0, were selected for use in the ENMs [37, 38]. See Additional file 1 for details.

### Grid selection of climate variables

Three sentinel hospitals were selected for patient data collection. The hospitals were located in Shanghai (provincial district in China), Zhenjiang (municipal), and Danyang (county), and were denoted as Hosp1, Hosp2, and Hosp3, respectively [39]. The data included results of *Cryptosporidium* detection and long-term address information of patients with diarrhea. First, the permanent addresses were imported into Google satellite maps (http://www.gditu.net/) for conversion into latitude and longitude coordinates, and then loaded into ArcMap of the ArcGIS v10.8 (ESRI Inc., Redlands, CA, USA) [40]. A point distance analysis tool was used to obtain the straight-line distance from the residence of patients to the hospitals. The Shapiro–Wilk (SW) and Levene’s tests were performed in IBM SPSS Statistics v26 (IBM Corp., Armonk, NY, USA) to observe the data distribution characteristics and homogeneity of variance [41]. Finally, box violin distribution plots were generated using Hiplot v0.2.0 (TengyunBioTech Ltd., Shanghai, China) to visualize the results [42]. The procedure was aimed at assessing the feasibility of the location coordinates of health care facilities as occurrence points of *Cryptosporidium* and selection of the spatial scales of the variables.

### Occurrence point data collection

Two types of publicly available historical data were collected: (i) *Cryptosporidium*-related studies and reports in China were obtained from PubMed database (https://pubmed.ncbi.nlm.nih.gov/), ResearchGate database (https://www.researchgate.net/), the China National Knowledge Infrastructure (CNKI) (https://www.cnki.net/) database, the Wanfang Database (https://new.wanfangdata.com.cn/index.html), and the China Science and Technology Journal Database (http://www.cqvip.com) by searching the following keywords: *Cryptosporidium*, cryptosporidiosis, diarrhea pathogen, intestinal protozoa, intestinal parasite, China, and province names in China; duplicate reports were removed. The remaining articles were carefully reviewed to screen relevant articles on human-derived *Cryptosporidium* or cryptosporidiosis in China. Articles (such as reviews and some basic research) from which reliable investigation periods and locations could not be extracted were excluded. To enhance the prediction level of models, *Cryptosporidium* survey data were collected from countries neighboring China from the PubMed database. Similar search and inclusion criteria were used in this section, excluding China being replaced with the other countries. Subsequently, if the sampling site was a hospital or another health care institution, and *Cryptosporidium* infection in patients attending the hospital was confirmed, the location coordinate of the institution was included as a *Cryptosporidium* occurrence point. If a *Cryptosporidium* infection was detected through field investigation, the coordinate of the investigation site was collected as an occurrence point. Latitude and longitude information and raster information of environmental factors were analyzed using ENMTools v1.4.4 to eliminate redundant points and to reduce their impact on model accuracy and reliability [43]. The cleaned data were imported into ArcMap to map the point distribution and case report areas of *Cryptosporidium* in China.

### Bioclimatic variable screening

Correlation between occurrence points and environmental variables potentially exists in the data. If the collinear variables are not screened, it can lead to model over-fitting [44]. In the present study, due to the variable interpretation requirement, the contribution value of each variable and the correlation coefficient between variables are considered comprehensively to exclude the collinear variables, instead of the principal component analysis method [45]. The percentage contribution (PC) of environmental factors to the model prediction results was assessed using the Jackknife method [9]. Subsequently, variable information for each point was extracted using ArcMap and imported into IBM SPSS Statistics 26 [41] to calculate the Pearson Correlation Coefficients (PCCs) among the variables. The contribution point plots and correlation heat maps of environmental variables were plotted using Hiplot v0.2.0 [42]. Among the variables with absolute correlation coefficients |r|≥ 0.8, only the variable with the largest contribution was retained [9].

### ENMs evaluation and selection

In the present study, an aggregate of four ENMs was tested. Maxent v3.4.3 (American Museum of Natural History, New York, USA) was used for the Maxent model [46], DIVA-GIS v7.5.0 was used for the BIOCLIM and Domain models [47], and DesktopGarp v1.1.6 was used for the Garp model [48]. The previously screened effective *Cryptosporidium* occurrence points were divided into two datasets: one had coordinate points collected from literature and imported for model construction, and another extracted from sentinel hospitals for use in evaluating model effectiveness. All the data, variables, and set parameters were maintained at default when calculating the four models, and predictions by each model were repeated 10 times. Finally, the test data and simulation results were imported into Niche Analysis v3.0 to plot the Receiver Operating Characteristic (ROC), Kappa, and True Skill Statistic (TSS) curves [49], and to further calculate the area value under ROC curve (AUC, the closer this value is to 1.00, the more accurate the results predicted by the model), maximum Kappa value (the closer this value is to 1.00, the better the consistency of the model), and TSS value (the closer this value is to 1.00, the more excellent the discrimination ability of the model) [50–52]. Scatter plots were drawn using Hiplot v0.2.0 [42].

### Parameter optimization of model

The two parameters that significantly impact model prediction results are Feature Combination (FC), including linear (L), quadratic (Q), hinge (H), product (P), and threshold (T), and Regularization Multipliers (RM) from 0.5 to 4.0 [53–55]. In the present study, the RM values were set from 0.5 to 4.0, with a 0.5 increase for each simulation. FC was simulated using seven combinations: H, L, LPQ, LQ, LQH, LQHP, and LQHPT. The final simulation results were evaluated using ROC and information criterion (IC). The ROC evaluation indexes include the AUC during model testing (AUC_Test_, larger values indicate better effects), which evaluates model performance under different threshold conditions, and the AUC difference between the training and test datasets (AUC_Differ_, lower values indicating greater gains), which evaluates the risk of overparameterization. The IC evaluation indexes were used to measure model complexity and goodness of fit, which include the sample size-corrected Akaike IC (AICc) and the Bayesian IC (BIC), and lower values suggest better balance between goodness of fit and reduced free parameters [53]. Matrix plots were visualized using Hiplot v0.2.0 [42].

### Effects of environmental variables on *Cryptosporidium* distribution

*Cryptosporidium* occurrence points were extracted from surveys conducted between 1986 and 2010, and were imported into the Maxent software. Historical climate data from CHELSA v2.1 (for the 1981‒2020 period) were selected. Climate variables and parameters were set according to the results of previous steps. Twenty-five percent of the random points were selected as the test data. The maximum number of background points was set as 10,000, the model was bootstrapped 100 times, and the results were obtained in cloglog format, a more effective format in explaining the prediction omission rate, to enable the calculation of the response curves of the variables used for modeling [56].

### Habitats currently suitable for *Cryptosporidium* in China

Based on the simulation results of data from 1986 to 2010, climate data layers from 2011 to 2040 were projected onto the model with the largest AUC value to predict the potential distribution of *Cryptosporidium* under current climate conditions. The occurrence points from 2010 to 2021 were imported into the prediction results as the test set to evaluate the effectiveness of the model. The final prediction results were imported into ArcGIS v10.8 [45], which was used to extract predicted *Cryptosporidium* maps within China. The average pixel values (Cloglog values) in the local spatial range were extracted from the county-level, city-level, and provincial-level areas to analyze current *Cryptosporidium* distribution in China.

### Potential distribution of *Cryptosporidium* in future

Based on the simulation results using climate data from 1981 to 2010, the future bioclimate variables provided by two climate data databases (WorldClim and CHELSA) were analyzed separately. The geographical areas of suitable habitats were calculated using ArcGIS v10.8 [40]. The areas were mapped using Hiplot v0.2.0 [42]. Chi-square (*χ*^2^) tests were performed to test differences in distribution areas during different periods using IBM SPSS v26 [41], and *P*-values were corrected using the Bonferroni method. The standard deviation of the predicted cloglog values of suitable area over four periods (1981‒2010, 2011‒2040, 2041‒2070, and 2071‒2100) were calculated using ArcGIS v10.8 [40] to analyze the degrees of variation in different areas. In addition, differences in predictions between two adjacent time periods were calculated and used to analyze changes in suitable *Cryptosporidium* habitats.

## Results

### Spatial scale of climate variable on the map

**Fig. 1:**
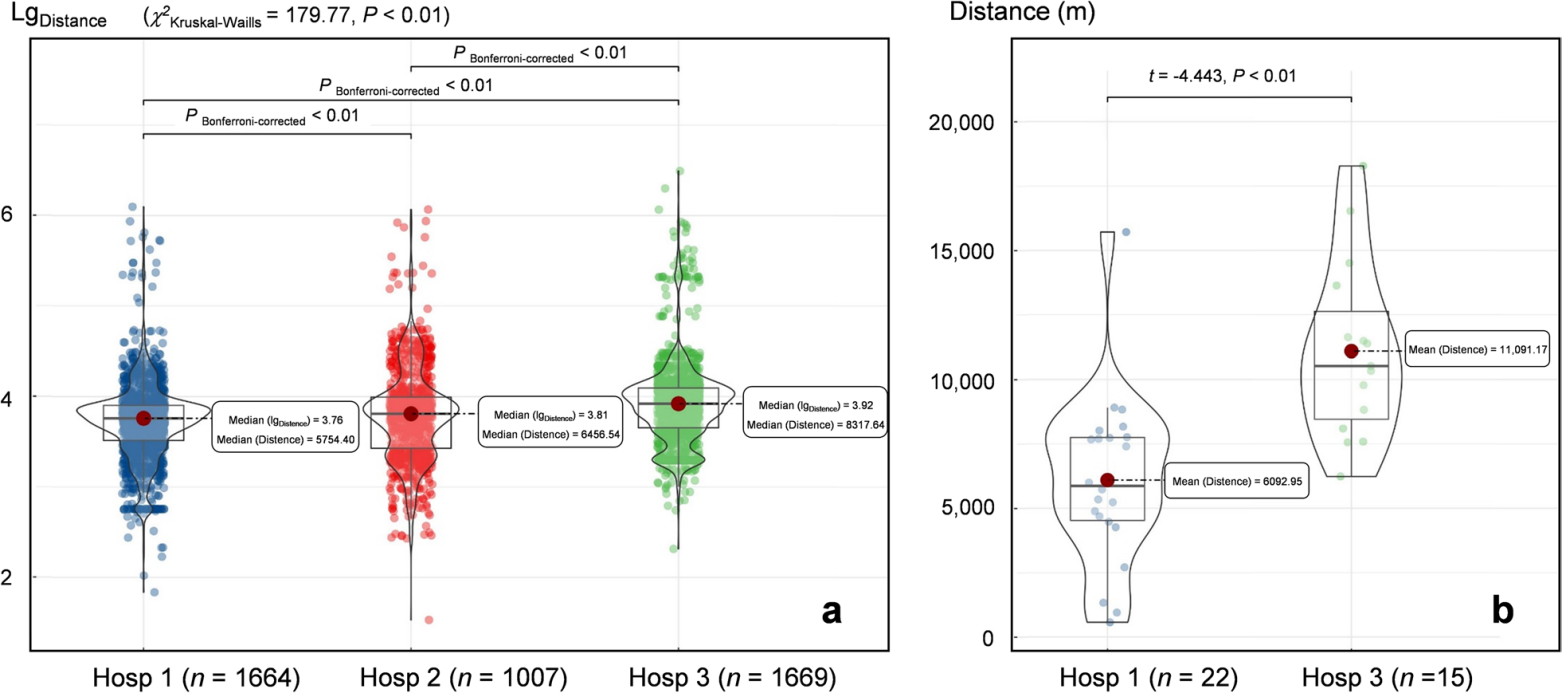
Box-violin diagram based on straight-line distance between patient residences and hospital visited. **a** Logarithmic distance between diarrhea patient residences and three hospitals. **b** Distances of travel of *Cryptosporidium*-infected patients during visits to Hosp1 and Hosp3 (no *Cryptosporidium* infection was detected in patients with diarrhea at Hosp2)

The straight-line distance data between the place of residence and the hospital selected by diarrhea patients did not follow a normal distribution (*W* = 0.121, *P* < 0.01) and exhibited heterogeneous variance (*L* = 5.213, *P* < 0.01), potentially because the patients included travelers from other places or temporary visitors. Hence, the median of logarithmic distance was selected to describe the data as a whole. As shown in Fig. 1a, using hospitals as the center points, the median distances for patients seeking medical advice were 5754.40 m (95% *CI*: 5596.75‒6007.88), 6456.54 m (95% *CI*: 6098.82‒6806.41) and 8317.64 m (95% *CI*: 7974.43‒8841.42) for Hosp1, Hosp2 and Hosp3, respectively. In contrast, distance data from 37 *Cryptosporidium*-infected patients were normally distributed (*W* = 0.966, *P* = 0.320) and exhibited homogeneous variance (*L* = 5.213, *P* < 0.01). Therefore, the average distances were 6092.95 m (95% *CI*: 4625.06‒7560.84) and 11,091.17 m (95% *CI*: 9190.44–12,991.90) for Hosp 1 and Hosp 3, respectively (Fig. 1b). The results indicate that the variable raster base map of 5 min (approximately 9 km × 9 km) can accommodate environmental information, with the hospital as the center point, and ensure prediction accuracy.

### Data collection and variable screening

In the present study, a total of 474 coordinate points related to the distribution of *Cryptosporidium* were extracted from the 243 collected research articles (see Additional file 2) and detection data of sentinel hospitals. After eliminating redundant data, 377 valid points were incorporated into the 5-min variable raster, including 228 points within China and 149 points within neighboring countries (see Additional file 3). To match the time nodes of periodic climate data obtained from the WorldClim and CHELSA databases, all points were classified into 154 points reported during 1986‒2000, 266 points reported during 1986‒2010, and 111 points reported during 2011‒2021, based on the survey date. *Cryptosporidium*-infected individuals were reported in 105 municipal administrative regions in 29 provincial administrative regions in China. In addition, to safeguard the privacy of participants, the specific addresses and coordinates of infected individuals were not included. Instead, only the area information are described in Additional file 4 and Additional file 5. After considering the average percentage contribution, and excluding possible collinearity of 19 bioclimatic variables observed, 10 variables were input in the simulation model, namely bio13 (precipitation of wettest month), bio1 (annual mean temperature), bio18 (precipitation of warmest quarter), bio5 (maximum temperature of warmest month), bio15 (precipitation seasonality), bio19 (precipitation of coldest quarter), bio8 (mean temperature of wettest quarter), bio2 (mean diurnal range), bio3 (isothermality), and bio14 (precipitation of driest month), which included five temperature-related variables and five precipitation-related variables (see Additional file 6).

### Model effectiveness

**Fig. 2:**
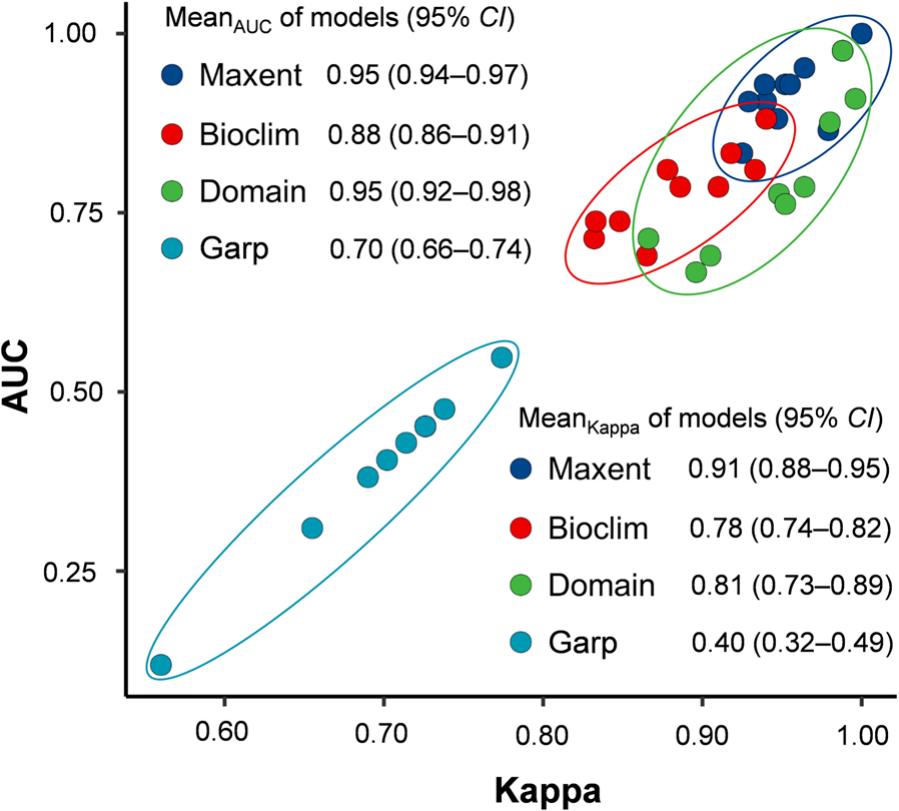
Scatter plots of AUC and maximum kappa values for four ecological niche models. AUC is the area under curve of receiver operating characteristic; Maxent is maximum entropy model; Bioclim is bioclimate model; Domain is domain model; Garp: the model based on genetic algorithms for rule-set production

Among the four modeling approaches tested in the present study, the Maxent, BIOCLIM, and Domain models achieved the maximum TSS value of 1.00 for each run, whereas the mean of the maximum TSS values for the Garp model was 0.95 (95% *CI*: 0.92‒0.97). As shown in Fig. 2, the Maxent model outperformed the other three models in prediction accuracy (0.95 AUC value with a range of 95% *CI*: 0.94‒0.97) and consistency (0.91 maximum Kappa value with 95% *CI* from 0.88 to 0.95).

### Model optimization and construction

**Fig. 3:**
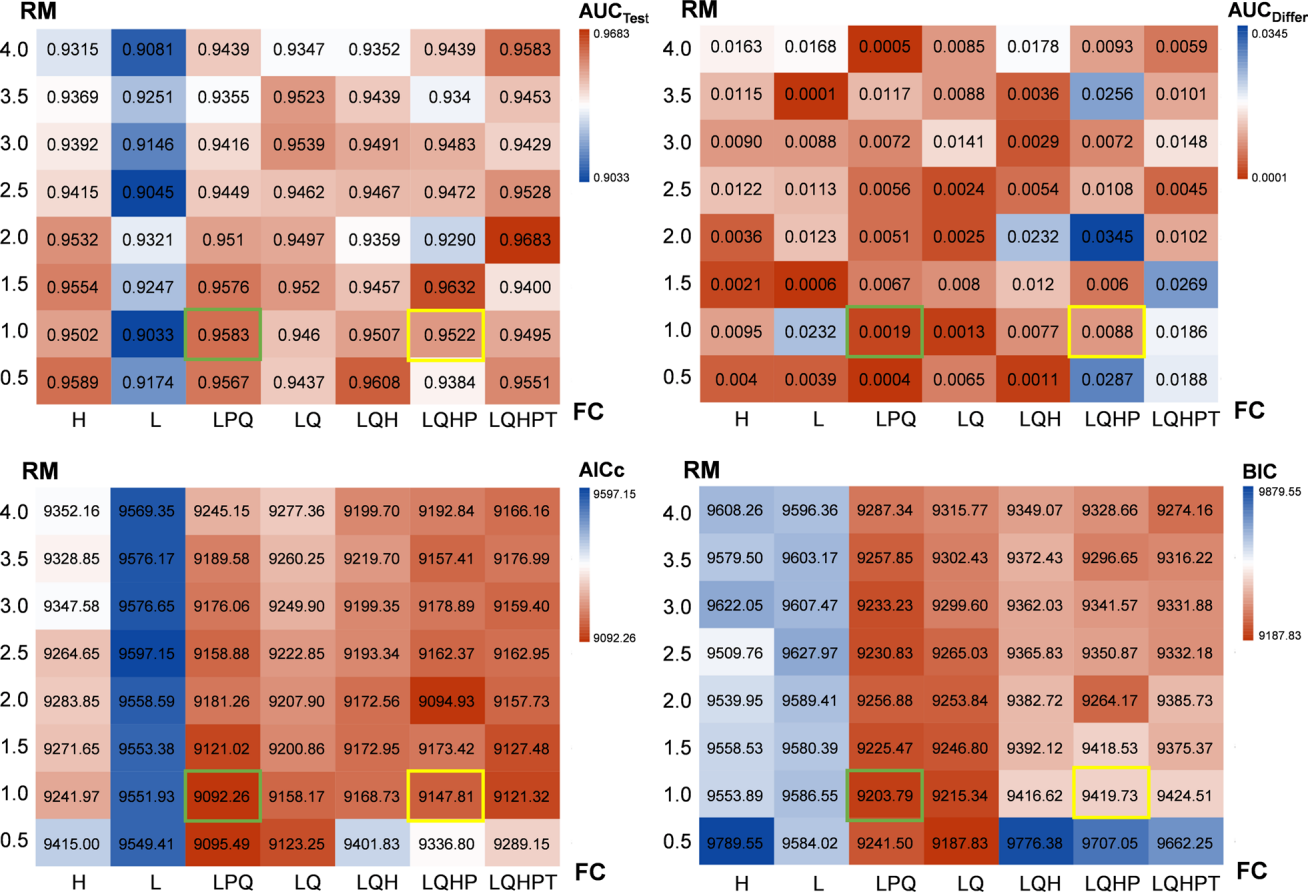
AUC_Test_, AUC_Differ_, AICc, and BIC matrix heat maps of models with different Regularization Multipliers (RM) parameters on Y-axes and Feature Combination (FC) parameters on X-axes in Maxent model. AUC_Test_ refers to the AUC (area under the receiver operating characteristic curve) obtained by the ENM in the testing procedure; AUC_Differ_ refers to the AUC difference between the training operation and the test operation; AICc: corrected Akaike information criterion; BIC: Bayesian information criterion

Two combinations of each index value ranked in the top 10 out of 56 parameter combinations of FC and RM were preferred, namely LPQ-0.5 and LPQ-1.0. Subsequently, the combination with superior AUC_Test_ and AICc values, namely LPQ-1.0, was selected (Fig. 3) to construct the Maxent model, and the simulation results with an average AUC of up to 0.939 ± 0.005 were finally obtained (Additional file 7). Therefore, compared with the optimized parameter settings (LPQ-1.0), the default model parameter setting (LQHP-1.0) did not yield optimal simulation results in the present study.

### Environmental effects on *Cryptosporidium* distribution

**Fig. 4:**
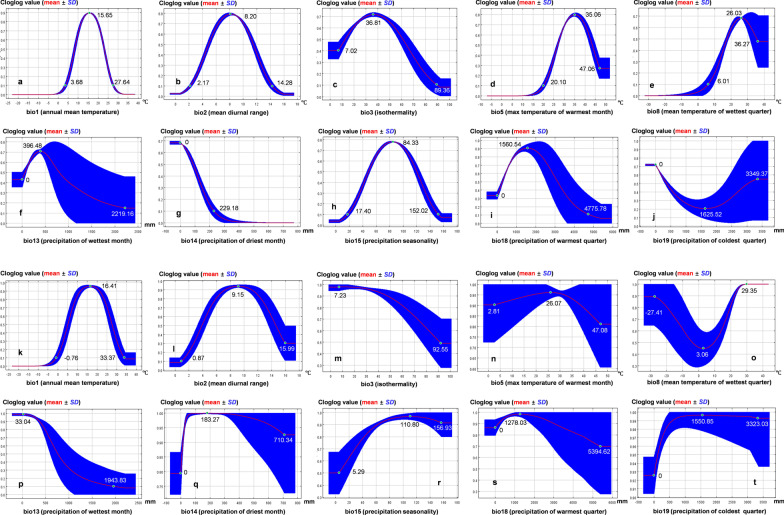
Response curves of 10 environmental variables included. **A–J** Independent response curves for environmental factors when all other environmental variables remain at their mean sample values. **K-T** Response curves for environmental variables used in model construction

The contributions of bio1, bio2, bio8, and bio18 to the model all exceeded 10%, and the cumulative contribution of the four factors was > 80%. Based on the prediction results, a cloglog value of 0.1 was set as the threshold for suitable *Cryptosporidium* habitats. When all other environmental variables were maintained at their mean values, the independent environmental factor response curves showed that the lowest annual mean temperature (bio1) at which *Cryptosporidium* could stably survive was 3.68 °C. Furthermore, the probability of survival increased with an increase in temperature. The maximum survival gain was obtained at 15.65 °C, with further temperature increases decreasing survival. When the temperature was higher than 27.64 °C, the habitat was no longer suitable for *Cryptosporidium* survival (Fig. 4a). When the bio1 was input into the model for comprehensive consideration, the survival temperature range was broader (− 0.76–33.37 °C) and the optimal response peak increased to 16.41 °C (Fig. 4k). The mean diurnal range represented by bio2 was suitable for *Cryptosporidium*, at 2.17–14.28 °C, with an optimum temperature of 8.20 °C (Fig. 4b). However, other variables in the model may compensate for the limitations of this variable for species distribution to some extent, thereby expanding the suitable temperatures to 0.87–15.99 °C and increasing the optimum temperature to 9.15 °C (Fig. 4l). The minimum temperature at which *Cryptosporidium* could stably survive during the season with the highest humidity (bio8) was 6.01 °C and the optimum survival environment was 26.03 °C (Fig. 4e); however, the variable does not limit *Cryptosporidium* survival in the comprehensive assessment of the model (Fig. 4o). Considering the specific heat capacity of water and the water-borne transmission of *Cryptosporidium*, the effects of temperature variation on the probability of *Cryptosporidium* survival may be offset by humidity; therefore, this result is reasonable. A similar variable is bio18, in which the maximum precipitation at which *Cryptosporidium* can stably survive during the warmest season is 4775.78 mm (Fig. 4i). After considering the interaction of each variable, *Cryptosporidium* could survive in the extreme conditions of bio18 (Fig. 4s), probably because warmer temperatures enhance *Cryptosporidium* reproduction and dispersal, thus offsetting forced oocyst migration caused by high precipitation. Although the extreme conditions are breached, the survival probability of *Cryptosporidium* is still affected by bio8 and bio18.

### Current potentially suitable habitats for *Cryptosporidium*

**Fig. 5:**
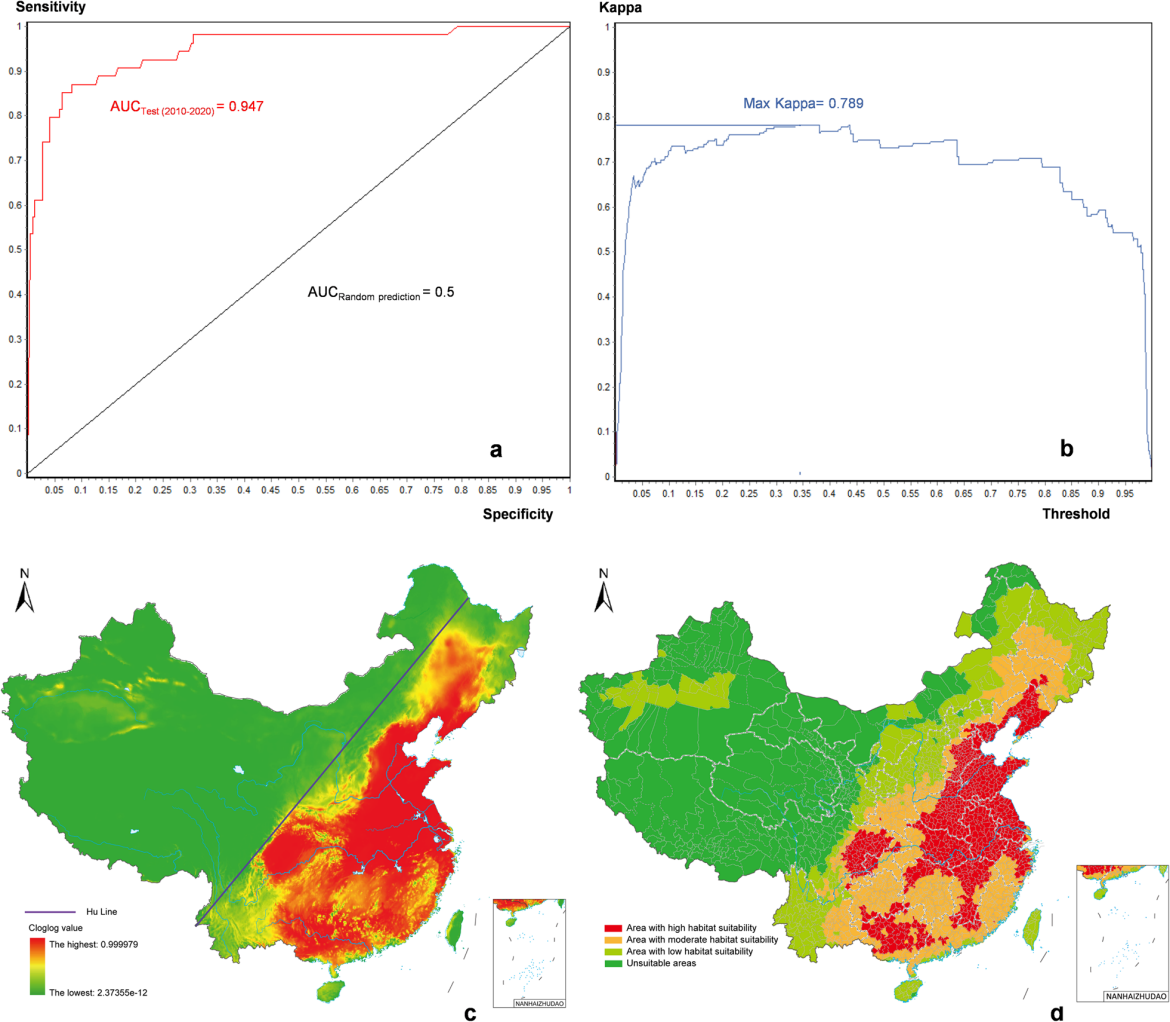
Predicted suitable habitats for *Cryptosporidium* in China at the present stage (2011‒2040). **a** Receiver Operating Characteristic (ROC) curves. **b** Kappa curves. **c** Heat maps of habitat suitability for *Cryptosporidium*. **d** Distribution of suitable habitats for *Cryptosporidium* by county-level administrative regions

The simulation prediction had an AUC of 0.947 and a maximum kappa value of 0.789 from 2011 to 2040, indicating that the model had good prediction effects, strong transferability, and high consistency, and can be used to describe and analyze current *Cryptosporidium* distribution. A cloglog value of 0.1 was set as the threshold for suitable *Cryptosporidium* habitats based on the results of the Maxent run. Considering *Cryptosporidium* may leave its native habitat via water flows and human activity, a more stringent threshold for areas with high habitat suitability (cloglog = 0.9) was selected. In addition, 0.1‒0.5 was set as the range for areas with low habitat suitability and 0.5‒0.9 was used for areas with moderate habitat suitability. The final results indicated that the areas with high habitat suitability for *Cryptosporidium* in China at this stage were primarily concentrated in Eastern and Central China including the entire regions of Jiangsu, Anhui, Shandong, Jiangxi, Henan and Hubei provinces, and Tianjin City, in addition to some cities in Hebei, Liaoning, Zhejiang, Hunan, Guangdong, Sichuan and Guangxi provinces. They were also concentrated in some districts and counties in the municipalities of Beijing, Shanghai, and Chongqing, and were sporadic in some regions in Jilin, Fujian, and Guizhou provinces. Non-suitable habitats included most of the Qinghai–Tibet Plateau, Hexi Corridor (most of Gansu Province), northern Heilongjiang Province, western Inner Mongolia Autonomous Region, and northern and southwestern Xinjiang Autonomous Region. Areas with moderate habitat suitability included areas outside the highly suitable and non-suitable habitats of all provincial administrative regions associated with high habitat suitability, also including southwestern Heilongjiang Province, southern Shanxi Province, central and southern Shaanxi Province, eastern Yunnan Province, southeastern Gansu Province, eastern Inner Mongolia Autonomous Region, Hainan and Taiwan islands, and Hong Kong Special Administrative Region. Areas with low habitat suitability were regions other than the aforementioned areas. In addition, all cities with high, moderate, and low habitat suitability for human-derived *Cryptosporidium* in China are listed in the supplementary file to support actual cryptosporidiosis control and prevention (see Additional file 8). In terms of geographical pattern, *Cryptosporidium* distribution areas were primarily located along the middle and lower reaches of the Yangtze River, the lower reaches of the Yellow River, and the Huai and the Pearl River Basin. Considering population development level and economic and social patterns, *Cryptosporidium* was mainly distributed below the Hu–Line (Heihe–Tengchong Line) [57], which is a region with high population density (see Fig. 5).

### Prediction of future *Cryptosporidium* distribution

**Fig. 6:**
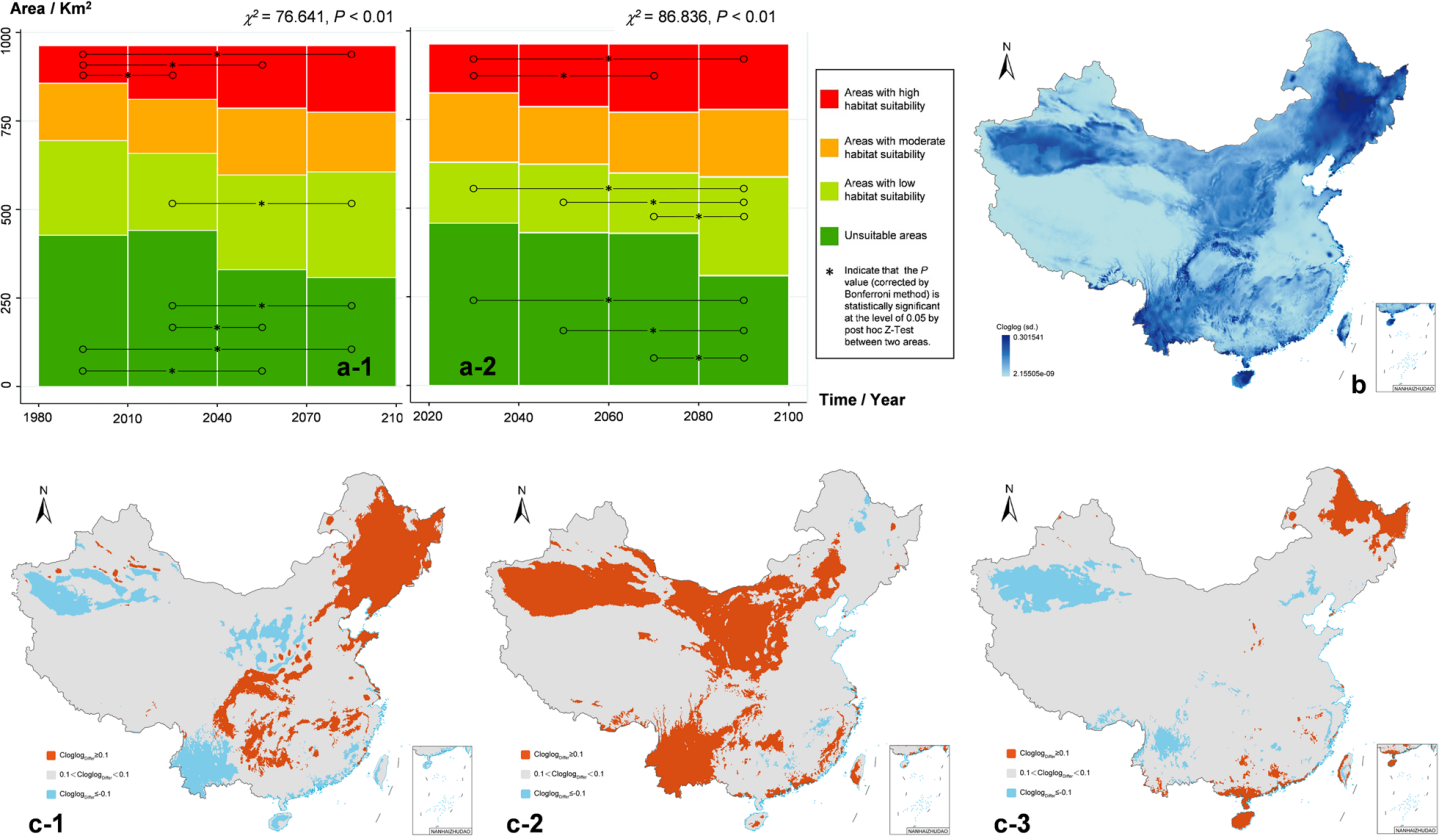
Predicted suitable habitats for *Cryptosporidium* based on future climate data. **a-1** Area maps of suitable habitats at each level based on climate data from the Climatologies at High Resolution for the Earth’s Land Surface Areas (CHELSA) database for four periods (1981‒2100). **a-2** Area maps of suitable habitats at each level based on climate data from the WorldClim database for four periods (2021‒2100). **b** The standard deviation of habitat suitability (Cloglog) based on CHELSA database simulations for four periods (1981‒2100). **c-1** Changes in habitat suitability based on the CHELSA database for the 2011‒2040 period relative to the 1981‒2010 period. **c-2** Changes in habitat suitability based on the CHELSA database for the 2041‒2070 period relative to the 2011‒2040 period. **c-3** Changes in habitat suitability (Cloglog) based on the CHELSA database for the 2071‒2100 period relative to the 2041‒2070 period. The absolute value of the cloglog difference of 0.1 was used as the threshold for habitat suitability in picture **c-1/c-2/c-3**

Under a climate change background, variation in the areas of habitats with moderate suitability was minor; shifts in the area of habitats were mainly reflected in decreasing areas of non-suitable habitats and expansion of areas of highly suitable habitats (Fig. 6a). The results indicate that *Cryptosporidium* will gain more suitable habitats in China in future (see Additional file 9 for details). As shown in Fig. 6b, variations in suitable habitats for *Cryptosporidium* from 1981 to 2100 were mainly concentrated in the northeastern, southwestern, southern, northwestern, and northern regions of China, with less changes in the eastern and Qinghai-Tibet Plateau areas. Furthermore, as illustrated in Fig. 6c-1, *Cryptosporidium* exhibited increased adaptation to climate in northeastern China, the coastal areas of northern China, and the edge of the Sichuan Basin from 2011 to 2040, relative to that from 1981 to 2010; conversely, reduced adaptation was observed in southwestern China, the coastal areas of southern China, and northwestern China, using an absolute value of the cloglog difference of 0.1 as the threshold for habitat suitability. Relative to that from 2011 to 2040, the predicted climate status from 2041 to 2070 is more favorable for *Cryptosporidium* survival in northern, northwestern and southwestern China, and along the coast of southern China (Fig. 6c-2); and areas with reduced survival probability were few. Figure 6c-3 shows the change in 2071‒2100 relative to the status in 2041‒2070: habitat suitability increased in Heilongjiang, Hainan, and coastal areas of Southern China, whereas *Cryptosporidium* survival probability decreased in northern Yunnan and central Xinjiang.

## Discussion

In the present study, a systematic ENM simulation for suitable *Cryptosporidium* habitats was conducted. Several key variables were tested and analyzed, in addition to data collection, screening, and collation; data applicability analysis; selection and calibration of environmental factor databases; and model selection, optimization, simulation, and evaluation. All defects and loopholes in the model simulation process were considered and avoided as much as possible. The final results indicated that the Maxent model with adjusted parameters returned the best simulation results with high evaluation indexes. The result confirms that ENMs can be applied to predict potential *Cryptosporidium* distribution, thereby providing insights and new methods for further research, and *Cryptosporidium* infection prevention and control. Moreover, when simulating parasitic infectious diseases using ENMs, it is necessary to assess which environmental variables may be meaningful using established information, while accounting for the biological characteristics of parasites, adopting reasonable spatial scales, performing spatial and temporal matching with species presence data, and ensuring data accessibility [23]. This is why we devote a lot of space in the Methods section to explaining the model running procedure.

According to the present study, at a macroscopic geographic scale, there are three areas with high suitability for human-derived *Cryptosporidium* in China: the plains in eastern China, the Pearl River Basin, and the Sichuan Basin. Notably, the areas are also high population areas. In addition, there is a clear unsuitable habitat zone, namely the Qinghai–Tibet Plateau, where the unique alpine climate limits *Cryptosporidium* survival and dispersal. The northeastern (Jilin and Heilongjiang Provinces), southwestern (Yunnan Province), and northwestern (Xinjiang Autonomous Region) regions are considerably affected by climate change, which may result in shifts in *Cryptosporidium* habitat suitability. It is critical to clarify that all projections of future climate change are based on simulations of historical data from 1981 to 2010, and only projections for the period from 2011 to 2040 can be verified using existing data. Therefore, the projections for the current period (2011–2040) are relatively reliable when compared to those of other periods (2041–2100) without test data. However, the fact that suitable *Cryptosporidium* habitats are expanding in China should not be overlooked. The species has gained survival benefits from climate change and may become widespread in future. Original parasite transmission dynamics may be disrupted and reshaped during climate change events. In addition, ongoing genetic evolution will lead to the emergence of subspecies and genetic subtypes that are adaptable to new environments and have increased potential for host switching [58]. Furthermore, new ecological fitting patterns may lead to more frequent transmission between humans and animals, which, in turn, would lead to unknown *Cryptosporidium* transmission risk in future, and make forward-looking research challenging. Therefore, continuous climate monitoring, dynamic “*Cryptosporidium*–environment” fitting studies, and time- and place-specific prevention, control, and planning activities could facilitate coping with the potential threat. In addition, seasonal variation in *Cryptosporidium* prevalence in different regions within China is irregular [6], which is also observed in India [59]. Identifying suitable habitats for *Cryptosporidium* may help explain this phenomenon; for example, the time required to reach suitable environmental conditions for *Cryptosporidium* spread is not consistent between high and low latitude or altitude areas, leading to uncertainty in the seasonal factors driving *Cryptosporidium* infection. Such inter-regional diversity is normal in a country as vast as China.

Areas with high habitat suitability provide conditions suitable for the survival of *Cryptosporidium*; therefore, the public health risk caused by cryptosporidiosis is relatively high in such areas. It may result in *Cryptosporidium* outbreaks even if a small number of infectious agents (infected individuals) are not controlled in time. Therefore, in such areas, a high degree of sustained monitoring is necessary. Cryptosporidiosis is also prevalent within areas with moderate habitat suitability. However, the periods suitable for *Cryptosporidium* survival may not be perennial in such areas; instead, the transmission risk of cryptosporidiosis may be significantly higher during warm and humid periods than in the other periods. In contrast, in areas with low habitat suitability, the survival space for *Cryptosporidium* is compressed; cryptosporidiosis transmission and infection may occur in months when conditions are suitable, with a low probability of outbreaks. Notably, *Cryptosporidium* infections may also occur in areas with unsuitable habitats, for example, when pathogens are carried into an area by travelers, with small-scale transmission potentially occurring by direct contact or indirect contact over a short period. When discussing the actual risks of cryptosporidiosis outbreak, practical factors, such as local population density, public health services and numbers of animal hosts should also be taken into account [60]. Therefore, the results of the present study based on models do not indicate the probability of finding *Cryptosporidium* species (oocyst) in a given location but provide a habitat suitability index (i.e., habitat quality for species survival and persistence) [9]. The results reflect whether the environmental conditions in a region are favorable for *Cryptosporidium* survival and spread when the pathogen is present, from which the likelihood of prevalence and outbreaks can be inferred.

Cryptosporidiosis is endemic globally and is among the most extensively distributed intestinal parasites, with an overall global prevalence of approximately 7.6% (95% CI: 6.9‒8.5) [4]. From 2009 to 2017, 444 outbreaks were reported within the United States alone, resulting in 7465 infections, 287 hospitalizations, and one death. The availability of such detailed epidemiological statistics was made possible by the inclusion of cryptosporidiosis in the National Outbreak Reporting System (USA), which has collected nationwide information on cryptosporidiosis since 1995 [61]. The system has been a tremendous resource for research on cryptosporidiosis risk factors, modes of transmission, and development of prevention and control programs [61]. In England and Wales, the Health Protection Agency and Infection Centers report epidemiological information and test results in a pyramidal structure to national surveillance centers to track the sources and transmission routes of cryptosporidiosis [62]. In France, the ANOFEL (French association of medical parasitologists) *Cryptosporidium* National Network was established in 2004. The network covers most of France and consists of 38 hospital-based parasite laboratories, and collects information on cryptosporidiosis case reports and samples, and publishes annual analyses to provide public health authorities with information on human cryptosporidiosis incidence and epidemiology [63]. In China, Feng et al. reported a cryptosporidiosis outbreak caused by possible intra-hospital infection and transmission that lasted more than 14 months (November 2007‒December 2008) in Shanghai, with up to 102 patients confirmed positive for *Cryptosporidium* [64]. Due to a lack of routine *Cryptosporidium* surveillance procedures, the initial source of the infection could not be identified [64]. Therefore, establishing and improving surveillance networks is critical for cryptosporidiosis control. We propose starting with highly suitable habitats, continuously enriching the database, updating the nodes of the surveillance network, and expanding the network to areas with moderate habitat suitability, to provide data support for early warning for *Cryptosporidium* outbreaks, traceability, prevention, and control.

*Cryptosporidium* infection was reported in 2.97% of subjects (5933/200,054) in an analysis of a 30-year period from 1987 to 2017 conducted in China [6]. Different prevalence rates of *Cryptosporidium* were observed in different age groups, with 2.56% in children under 5 years of age, 3.68% in adolescents, and 1.89% in adults, 6.57% in HIV-positive/AIDS patients, 4.89% in HBV-positive patients, 47.79% in cancer patients, and 24.14% among drug users [6]. Population characteristics of infection are consistent with global statistics: minors and people with dysfunctional, depressed, and compromised immune systems are the priority populations for *Cryptosporidium* infection, and should be the focus of surveillance efforts [4]. *Cryptosporidium* oocysts can contaminate drinking, recreational, and agricultural water following contact with infected humans or animals, leading to *Cryptosporidium* spread and transmission [65]. Therefore, detecting *Cryptosporidium* oocysts in water bodies has become an indicator for the assessment of water quality in many countries and regions [65]. Additionally, *Cryptosporidium* is among the most important foodborne parasites, and is usually transmitted through the fecal–oral route [1]. In recent years, outbreaks of foodborne *Cryptosporidium* have been associated with shifts in consumer dietary habits in favor of fresh produce. Since fresh produce is not sterilized or treated at high temperature, it is highly likely a vector for *Cryptosporidium* transmission. *Cryptosporidium* has been detected in foods such as fruits and vegetables in several countries globally, with an overall positivity rate of 6.0% (375/6210) [66]. Another characteristic of *Cryptosporidium* is its wide range of host animal species. Over 260 species, including mammals, reptiles, birds, amphibians, and fish, are potential hosts of *Cryptosporidium* spp., and more than 155 mammalian species are hosts of *C. parvum*. *Cryptosporidium* is adapting and evolving to infect more diverse hosts and is becoming a major zoonotic problem [67]. Vermeulen et al. calculated the global *Cryptosporidium* emission load from livestock and showed that approximately 3.2 × 10^23^ oocysts are discharged from livestock feces, with the highest contribution from cattle, followed by chicken and pigs [68]. Based on the aforementioned considerations, the One Health approach that includes the “human–animal–water–food–other interface” should be promoted in the integrated infectious disease surveillance networks to be established for cryptosporidiosis and other neglected tropical diseases [7].

In line with the One Health concept, it is necessary to develop a comprehensive public health approach encompassing “parasite–host–environment” for cryptosporidiosis prevention and control. In such a strategy, real-time detection and surveillance should be conducted, information exchange and sharing between internal and external parties should be increased, and effort should be made to improve surveillance networks and reporting systems. In addition, interdisciplinary and inter-departmental prevention and control mechanisms should be proposed to prevent, diagnose, monitor, control, treat, and protect against cryptosporidiosis over a wider scope, and to better assess the risk of cryptosporidiosis outbreaks and minimize hidden public health safety risks. Meanwhile, progress in drug development and advancement in vaccine development remain critical for the containment of this important pathogen.

Considering the paucity of relevant research, we could only conduct spatial simulations over large geographic scales based on available information on cryptosporidiosis in China, relying on climate data to explore strategies and models for cryptosporidiosis prevention and control. However, in the context of specific control options, a small geographic scale and multidimensional simulations on *Cryptosporidium* transmission dynamics may provide more specific suggestions. Secondly, due to the lack of research on the molecular biology of *Cryptosporidium* in China, it is not yet possible to analyze the spatial ecological niche relationships among different species. Considering cross-species transmission between humans and animals, studies on overlapping and separation of spatial ecological niches for *Cryptosporidium* isolated from different hosts may provide information that could facilitate cryptosporidiosis prediction and early warning.

## Conclusions

The Maxent model can achieve sound results with regard to *Cryptosporidium* distribution prediction. In China, large areas are suitable for *Cryptosporidium* survival, which are mainly concentrated on the areas with high population densities. Key areas include the North China Plain, the Middle-Lower Yangtze Plain, and the Pearl River Basin. In contrast, the climate environment in the Qinghai–Tibet Plateau region is not suitable for *Cryptosporidium* survival and spread. In the context of future climate change, *Cryptosporidium* may gain more suitable habitats within China. Areas including northeastern, northwestern, and southwestern China and coastal regions require dynamic attention. Establishment of a national surveillance network in areas with suitable *Cryptosporidium* habitats could further elucidate the epidemiological trends and transmission patterns of cryptosporidiosis. The modelling information reported in the present study could facilitate the prevention and control of *Cryptosporidium* and other pathogenic intestinal protozoa.

## Supplementary Information

**Additional file 1.** Brief description of WorldClim and Climatologies at High Resolution for the Earth’s Land Surface Areas (CHELSA) database characteristics.

**Additional file 2.** Study and analysis flow chart.

**Additional file 3.** Maps of extracted *Cryptosporidium* presence data (occurrence points) in China and neighboring countries.

**Additional file 4.** Reference list of reports of human infected with *Cryptosporidium* in China.

**Additional file 5.** References for human cases of *Cryptosporidium* in countries neighboring China.

**Additional file 6.** Combined points graph of percentage contribution (PC) and plots of Pearson Correlation Coefficients (PCCs) for 19 bioclimatic variables.

**Additional file 7.** Predicted habitat suitability for *Cryptosporidium* based on historical data (1981‒2010). a-1) Predicted suitable habitats for *Cryptosporidium*. a-2) Standard deviation of prediction results (Cloglog value). b) Receiver Operating Characteristic (ROC) curves for predicted suitable habitats.

**Additional file 8.** Statistics of cities with high, moderate, and low habitat suitability for human-derived *Cryptosporidium* in China.

**Additional file 9. **Predicted habitat suitability for *Cryptosporidium* based on historical data of the 1971‒2100 period. a-e) Predicted results based on climate data from the WorldClim database for the 1971‒2000, 2021‒2040, 2041‒2060, 2061‒2080, and 2081–2100 periods. f-i) Predicted results based on climate data from the Climatologies at High Resolution for the Earth’s Land Surface Areas (CHELSA) database for the 1981‒2010, 2011‒2040, 2041‒2070, and 2071‒2100 periods.

## Data Availability

All the relevant data used and generated during this study are included in the manuscript.

## Abbreviations

AICc: Sample size corrected Akaike information criterion
AUC: Area under curve
BIC: Bayesian information criterion
Bioclim: Bioclimate analysis and prediction system
CMIP: Coupled Model Intercomparison Project
ENMs: Ecological niche models
FC: Feature combination
Garp: Genetic algorithm for rule-set production
GBD: Global burden of disease study
GCMs: Global climate models
CHELSA: Climatologies at high resolution for the earth’s land surface areas
IPCC: Assessment Report 6 of Intergovernmental Panel on Climate Change
Maxent: Maximum entropy
PC: Percent contribution
PCCs: Pearson correlation coefficients
RCPs: Representative concentration pathways
RM: Regularization multiplier
ROC: Receiver operating characteristic
SDMs: Species distribution models
SSPs: Shared socio-economic pathways
TSS: True skill statistic
WCRP: World Climate Research Program
WHO: World Health Organization
