# Supplementary material for: Cryptosporidiosis threat under climate change in China: prediction and validation of habitat suitability and outbreak risk for human-derived Cryptosporidium based on ecological niche models
Source: Infect Dis Poverty. 2023 Apr 11;12:35. doi: 10.1186/s40249-023-01085-0 (PMC10088348; doi:10.1186/s40249-023-01085-0)
Supplement: Supplementary file 1 — Additional file 1. Brief description of WorldClim and Climatologies at High Resolution for the Earth’s Land Surface Areas (CHELSA) database characteristics. [file 40249_2023_1085_MOESM1_ESM.docx]

**Additional file 1** Brief description of WorldClim and Climatologies at High Resolution for the Earth’s Land Surface Areas (CHELSA) database characteristics

| Feature | WorldClim | CHELSA |
| --- | --- | --- |
| Version | 2.1 | 2.1 |
| Time of latest data released | January 2020 | May 2021 |
| No. bioclimatic variables | 19^a^ | 76 |
| Periods of historical data | 1971–2000 | 1981–2010 |
| Periods of future data | 4 periods (2021–2040, 2041–2060, 2061–2080 and 2081–2100) | 3 periods (2011–2040, 2041–2070 and 2071–2100) |
| Sources of future data | IPCC6 & CMIP6 | IPCC6 & CMIP6 |
| No. SSPs of future data | 4 (126, 245, 370 and 585) | 3 (126, 370 and 585) |
| No. GCMs of future data | 23^b^ | 5 |
| No. spatial resolutions | 4 (30 secs, 2.5 mins, 5 mins, 10 mins) | 30 secs |
| Downscaling and calibration of data | Already completed | Set by user |

^a^ bio1 - annual mean temperature (℃), bio2-mean diurnal range (℃), bio3 – isothermality (bio2/bio7 × 100), bio4 - temperature seasonality (standard  deviation × 100), bio5 - maximum temperature of warmest month (℃), bio6 - minimum temperature of coldest month (℃), bio7 = temperature annual range (℃), bio8 - mean temperature of wettest quarter (℃), bio9 - mean temperature of driest quarter (℃), bio10 - mean temperature of warmest quarter (℃), bio11 - mean temperature of coldest quarter (℃), bio12 - annual precipitation (mm), bio13 - precipitation of wettest month (mm), bio14 - precipitation of driest month (mm), bio15 - precipitation seasonality (coefficient of variation), bio16 - precipitation of wettest quarter (mm), bio17 - precipitation of driest quarter (mm), bio18 - precipitation of warmest quarter (mm), bio19 - precipitation of coldest quarter (mm).

^b^ ACCESS-CM2, ACCESS-ESM1-5, BCC-CSM2-MR, CanESM5, CanESM5-CanOE, CMCC-ESM2, CNRM-CM6-1, CNRM-CM6-1-HR, CNRM-ESM2-1, EC-Earth3-Veg, EC-Earth3-Veg-LR, FIO-ESM-2-0, GFDL-ESM4, GISS-E2-1-G, GISS-E2-1-H, HadGEM3-GC31-LL, INM-CM4-8, INM-CM5-0, IPSL-CM6A-LR, MIROC-ES2L, MIROC6, MPI-ESM1-2-HR, MPI-ESM1-2-LR, MRI-ESM2-0, UKESM1-0-LL, GFDL-ESM4, IPSL-CM6A-LR, MPI-ESM1-2-HR, MPI-ESM2-0, UKESM1-0-LL
