# Supplementary figures and images for: Cryptosporidiosis threat under climate change in China: prediction and validation of habitat suitability and outbreak risk for human-derived Cryptosporidium based on ecological niche models
Source: Infect Dis Poverty. 2023 Apr 11;12:35. doi: 10.1186/s40249-023-01085-0 (PMC10088348; doi:10.1186/s40249-023-01085-0)

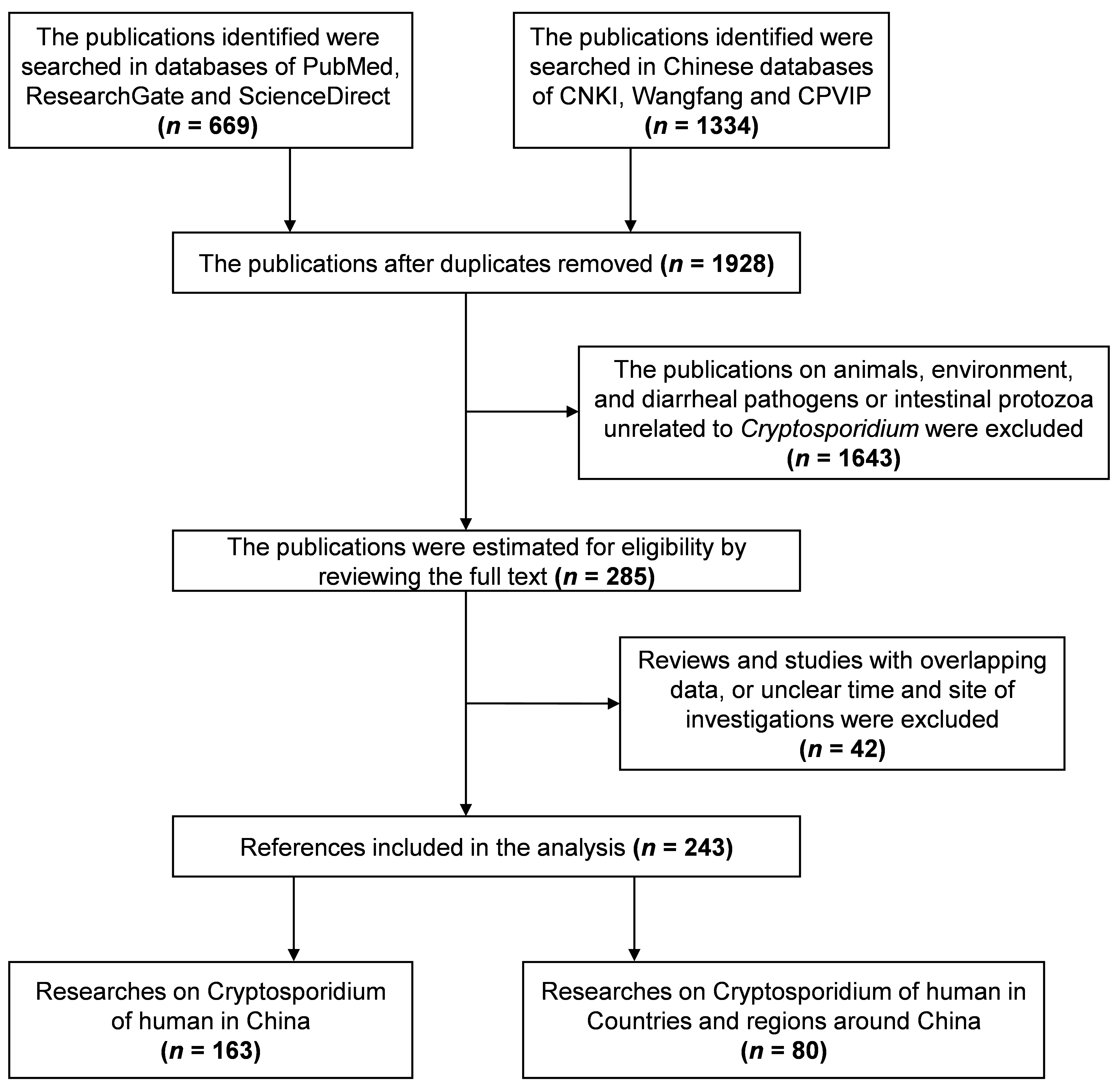

Supplement: Supplementary file 2 — Additional file 2. Study and analysis flow chart. [file 40249_2023_1085_MOESM2_ESM.tif]

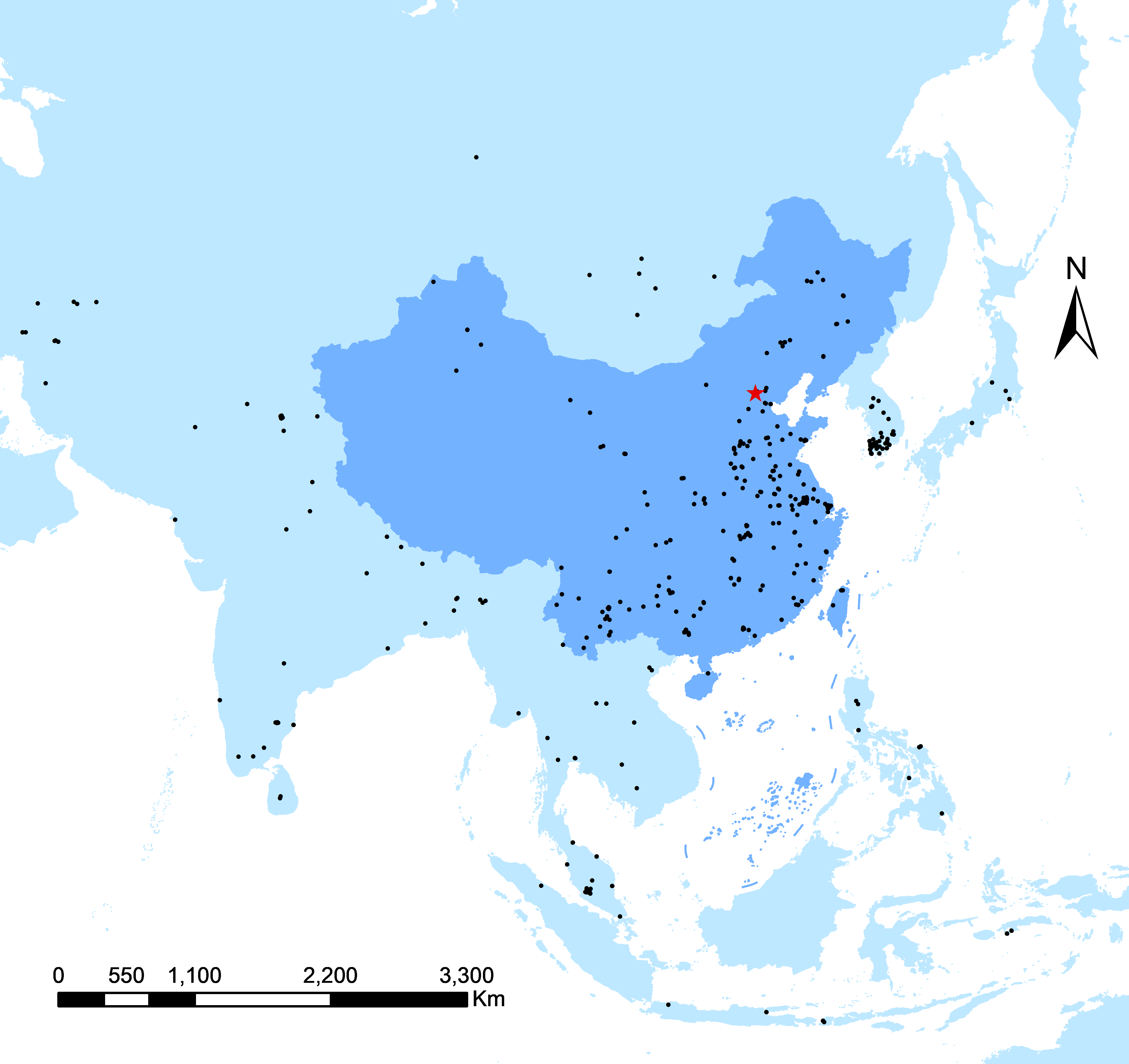

Supplement: Supplementary file 3 — Additional file 3. Maps of extracted Cryptosporidium presence data (occurrence points) in China and neighboring countries. [file 40249_2023_1085_MOESM3_ESM.tif]

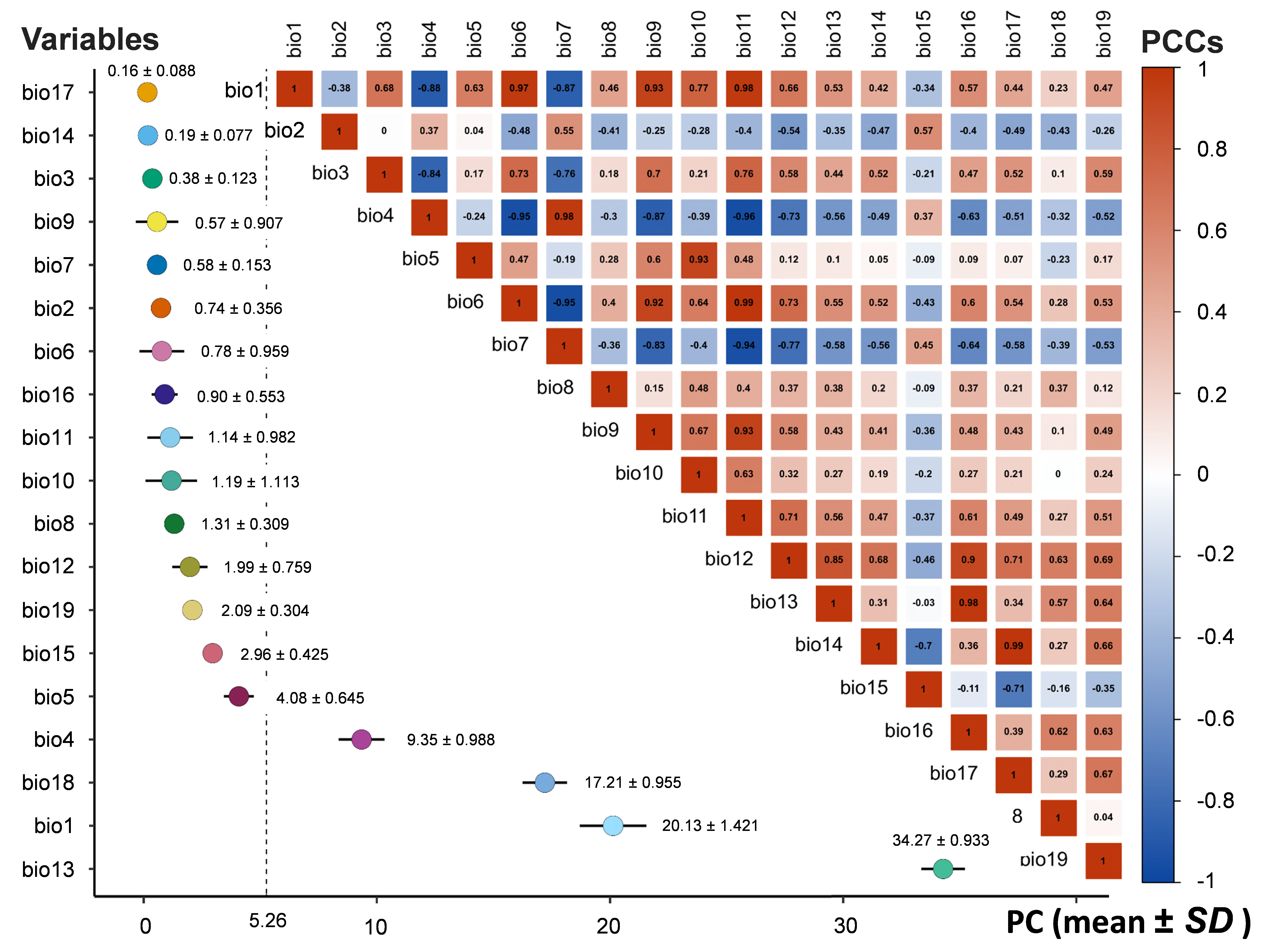

Supplement: Supplementary file 6 — Additional file 6. Combined points graph of percentage contribution (PC) and plots of Pearson Correlation Coefficients (PCCs) for 19 bioclimatic variables. [file 40249_2023_1085_MOESM6_ESM.tif]

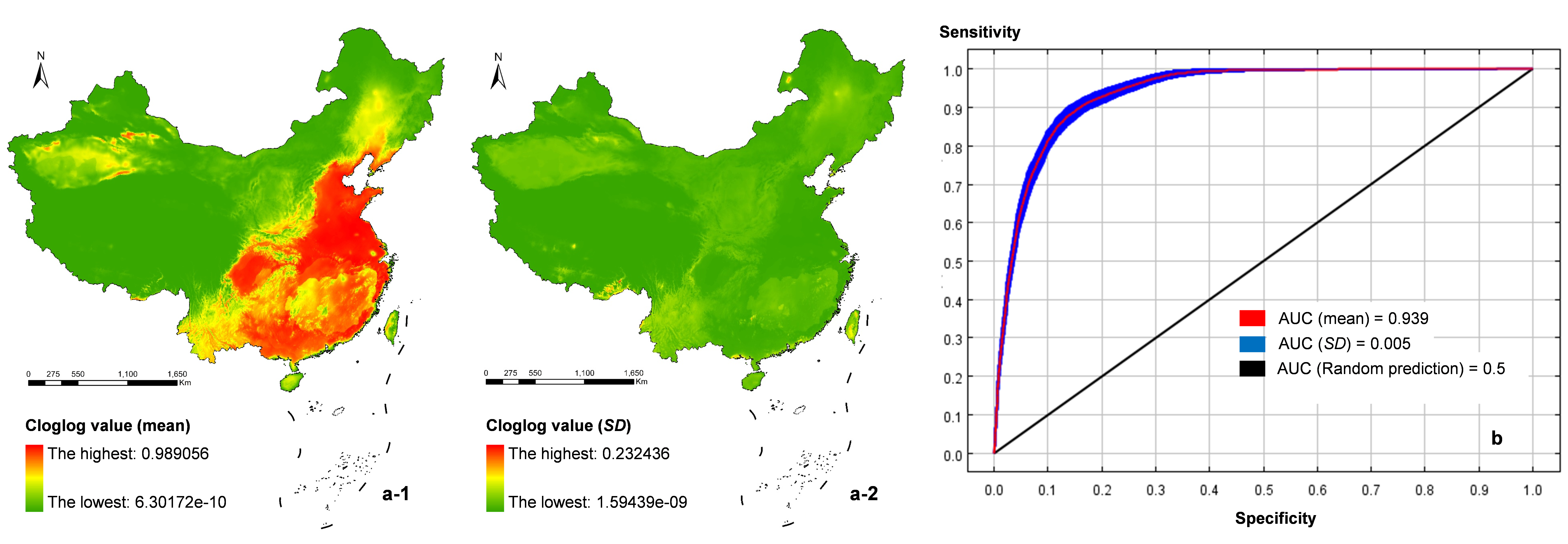

Supplement: Supplementary file 7 — Additional file 7. Predicted habitat suitability for Cryptosporidium based on historical data (1981‒2010). a-1) Predicted suitable habitats for Cryptosporidium. a-2) Standard deviation of prediction results (Cloglog value). b) Receiver Operating Characteristic (ROC) curves for predicted suitable habitats. [file 40249_2023_1085_MOESM7_ESM.tif]

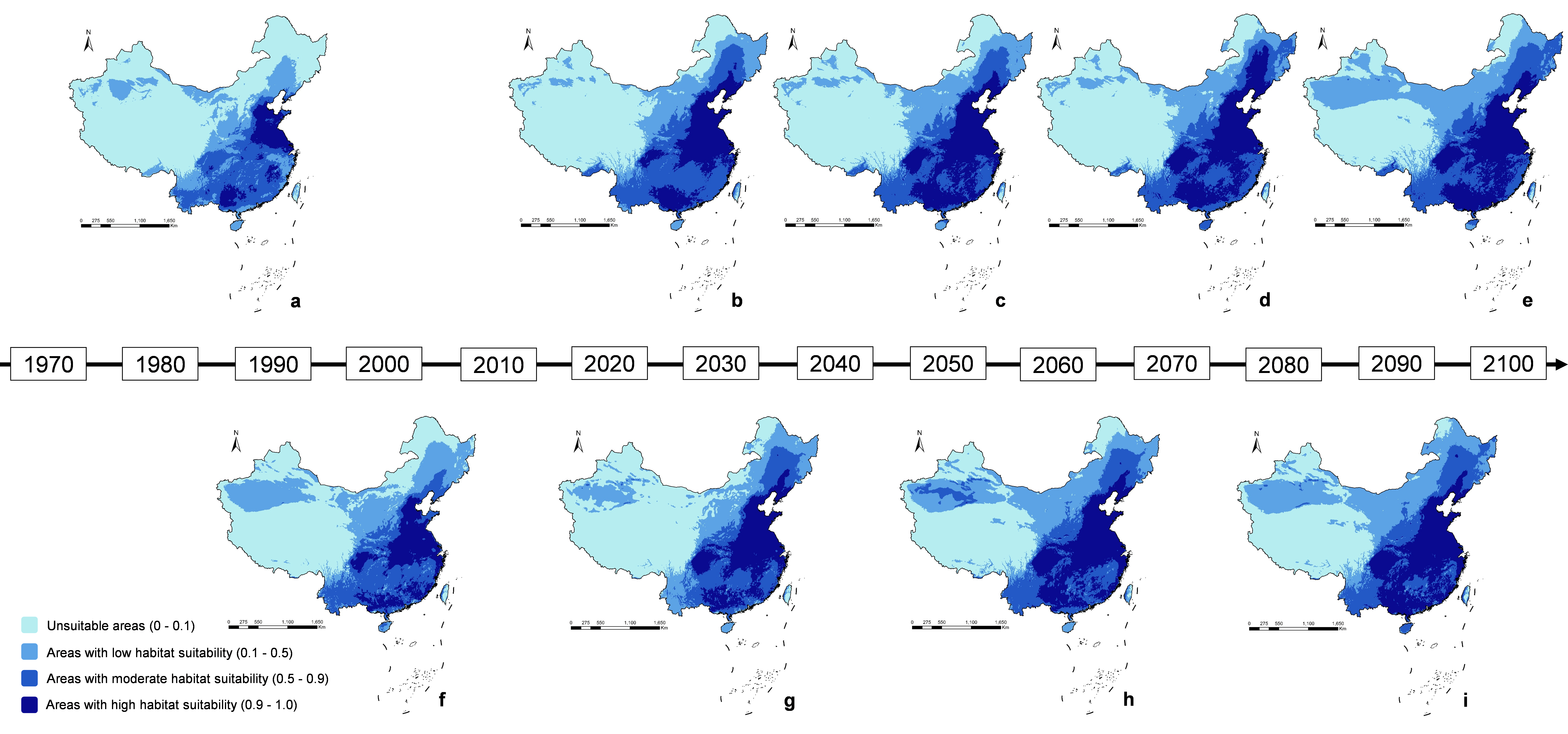

Supplement: Supplementary file 9 — Additional file 9. Predicted habitat suitability for Cryptosporidium based on historical data of the 1971‒2100 period. a-e) Predicted results based on climate data from the WorldClim database for the 1971‒2000, 2021‒2040, 2041‒2060, 2061‒2080, and 2081–2100 periods. f-i) Predicted results based on climate data from the Climatologies at High Resolution for the Earth’s Land Surface Areas (CHELSA) database for the 1981‒2010, 2011‒2040, 2041‒2070, and 2071‒2100 periods. [file 40249_2023_1085_MOESM9_ESM.tif]
