# Supplementary material for: Cryptosporidiosis threat under climate change in China: prediction and validation of habitat suitability and outbreak risk for human-derived Cryptosporidium based on ecological niche models
Source: Infect Dis Poverty. 2023 Apr 11;12:35. doi: 10.1186/s40249-023-01085-0 (PMC10088348; doi:10.1186/s40249-023-01085-0)
Supplement: Supplementary file 4 — Additional file 4. Reference list of reports of human infected with Cryptosporidium in China. [file 40249_2023_1085_MOESM4_ESM.docx]

**Additional file 3** Reference list of reports of human infected with *Cryptosporidium* in China

| Areas | Cities | Prevalence % | Detection method | Examined population | Investigation time | References |
| --- | --- | --- | --- | --- | --- | --- |
| Beijing City |  | 2.33 (7/30) | Molecular biology | HIV/AIDS patients | Before 2006 | ^[1]^ |
|  |  | 1.28 (30/2344) | Immunology | Diarrhea patients | 2004-2009 | ^[2]^ |
|  |  | 5.97 (4/67) | Pathobiology | HIV/AIDS patients with diarrhea | 2009-2011 | ^[3]^ |
|  |  | 8.80 (11/125) | Pathobiology | HIV/AIDS patients with diarrhea | 2012-2015 | ^[4]^ |
|  |  | 0.84 (5/592) | Pathobiology | Diarrhea patients | 2013-2014 | ^[5]^ |
| Tianjin City |  | 3 | Pathobiology | Case report | 1990 | ^[6]^ |
|  |  | 0.92 (11/1200) | Pathobiology | Diarrhea patients | 1991 | ^[7]^ |
|  |  | 10.45 (30/287) | Pathobiology | Diarrhea patients | 1998-1999 | ^[8]^ |
|  |  | 5 | Molecular biology | Case study | 1998-2000 | ^[9]^ |
|  |  | 0.79 (2/422) | Pathobiology | Diarrhea patients | 2014 | ^[10]^ |
| Hebei Province | Shijiazhuang | 4.62 (6/130) | Pathobiology | Diarrhea patients | 2008-2012 | ^[11]^ |
|  | Handan | 1.87 (2/107) | Pathobiology | Diarrhea patients | 1991 | ^[12]^ |
|  |  | 2.17 (106/4889) | Pathobiology | General population | 1991-1992 | ^[13]^ |
|  | Baoding | 6.04 (9/149) | Pathobiology | Diarrhea patients | Before 1997 | ^[14]^ |
|  |  | 2.90 (2/69) | Pathobiology | Diarrhea patients | 1997-1998 | ^[15]^ |
|  | Cangzhou | 1.00 (1/100) | Pathobiology | Diarrhea patients | 2018-2019 | ^[16]^ |
| Inner Mongolia Autonomous Region | Huhhot | 3.57 (5/140) | Pathobiology | Diarrhea patients | 1988 | ^[17]^ |
|  | Chifeng | 4.19 (42/1002) | Pathobiology | General population | 2012-2014 | ^[18]^ |
| Liaoning Province | Shenyang | 3.18 (9/283) | Pathobiology | Diarrhea patients | 2003-2004 | ^[19]^ |
| Jilin Province | Changchun | 3.18 (65/2046) | Immunology | Diarrhea patients | 2010 | ^[20]^ |
|  | Jilin | 14.29 (13/91) | Pathobiology | HIV/AIDS patients with diarrhea | 2014-2016 | ^[21]^ |
| Heilongjiang Province | Harbin | 1.39 (13/931) | Pathobiology | Diarrhea patients | 1991 | ^[22]^ |
|  | Qiqihar | 3.33 (11/330) | Pathobiology | Diarrhea patients | 1998-1999 | ^[23]^ |
|  |  | 15.52 (9/58) | Pathobiology | HIV/AIDS patients | 2014-2016 | ^[24]^ |
| Shanghai City |  | 2.35 (2/85) | Pathobiology | General population | 2004 | ^[25]^ |
|  |  | 1.62 (102/6284) | Molecular biology | Diarrhea patients | 2007-2009 | ^[26]^ |
|  |  | 5.33 (12/225) | Pathobiology | Diarrhea patients | 2009 | ^[27]^ |
|  |  | 9.17 (10/109) | Molecular biology | Diarrhea patients | 2011 | ^[28]^ |
|  |  | 0.07 (4/5939) | Pathobiology | Diarrhea patients | 2011-2013 | ^[29]^ |
|  |  | 2.21 (24/1087) | Molecular biology | Diarrhea patients | 2011-2015 | ^[30]^ |
|  |  | 13.49 (34/252) | Molecular biology | Diarrhea patients | 2012-2013 | ^[31]^ |
|  |  | 1.31 (37/2817) | Pathobiology | Diarrhea patients | 2013-2015 | ^[32]^ |
|  |  | 0.04 (3/6984) | Pathobiology | Diarrhea patients | 2015-2019 | ^[33]^ |
| Jiangsu Province | Nanjing | 1.29 (59/4582) | Pathobiology | Diarrhea patients | 1986-1988 | ^[34]^ |
|  |  | 3.18 (163/5124) | Pathobiology | Diarrhea patients | 1988-1989 | ^[35]^ |
|  |  | 0.79 (16/2018) | Pathobiology | General population | 1989 | ^[36]^ |
|  |  | 0.09 (1/1065) | Pathobiology | General population | 1989 | ^[37]^ |
|  |  | 1.02 (7/684) | Pathobiology | General population | 1989-1990 | ^[38]^ |
|  |  | 0.78 (7/896) | Pathobiology | Diarrhea patients | 2008 | ^[39]^ |
|  |  | 4.46 (9/202) | Pathobiology | Diarrhea patients | 2015-2016 | ^[40]^ |
|  | Wuxi | 1.37 (2/146) | Pathobiology | Diarrhea patients | 2015-2016 | ^[41]^ |
|  | Xuzhou | 5.22(12/230) | Pathobiology | General population | 1988 | ^[42]^ |
|  |  | 4.18 (20/478) | Pathobiology | Diarrhea patients | 1988 | ^[43]^ |
|  |  | 4.02 (46/1144) | Pathobiology | General population | 1989-1990 | ^[38]^ |
|  | Changzhou | 1.09 (18/1651) | Pathobiology | General population | 2013 | ^[44]^ |
|  | Suzhou | 1.20 (10/835) | Pathobiology | General population | 1989-1990 | ^[38]^ |
|  | Nantong | 0.32 (1/314) | Pathobiology | Diarrhea patients | Before 1991 | ^[45]^ |
|  | Lianyungang | 0.70 (5/718) | Pathobiology | General population | 1989-1990 | ^[38]^ |
|  | Huaian | 1.96 (13/662) | Pathobiology | General population | 1989-1990 | ^[38]^ |
|  | Yangzhou | 0.76 (8/1046) | Pathobiology | General population | 1989-1990 | ^[38]^ |
|  | Zhenjiang | 1.77 (28/1578) | Molecular biology | Diarrhea patients | 2011-2015 | ^[30]^ |
| Zhejiang Province | Hangzhou | 10.40 (57/548) | Pathobiology | Diarrhea patients | 1999 | ^[46]^ |
|  | Wenzhou | 5.66 (60/1060) | Pathobiology | Diarrhea patients | 1995-1996 | ^[47]^ |
|  |  | 1 | Pathobiology | Case report | 2001 | ^[48]^ |
|  | Quzhou | 32.16 (53/173) | Immunology | General population | 2005 | ^[49]^ |
| Anhui Province | Hefei | 1.20 (6/500) | Pathobiology | General population | 2001 | ^[50]^ |
|  | Huaibei | 1 | Pathobiology | Case report | 1987 | ^[51]^ |
|  |  | 1.82 (8/440) | Pathobiology | General population | 2001 | ^[50]^ |
|  | Suzhou | 1.57 (7/446) | Pathobiology | General population | 2001 | ^[50]^ |
|  |  | 2.96 (9/304) | Pathobiology | General population | 2014-2015 | ^[52]^ |
|  | Bengbu | 1.15 (4/349) | Pathobiology | General population | 2001 | ^[50]^ |
|  | Fuyang | 1.80 (9/500) | Pathobiology | General population | 2001 | ^[50]^ |
|  |  | 8.28 (21/302) | Pathobiology | HIV/AIDS patients | 2008 | ^[53]^ |
|  |  | 4.44 (32/720) | Pathobiology | General population | 2008 | ^[53]^ |
|  |  | 1.04 (5/480) | Pathobiology | General population | 2014-2015 | ^[52]^ |
|  | Huainan | 1.38 (13/939) | Pathobiology | General population | 2001 | ^[50]^ |
|  |  | 5.56 (46/827) | Pathobiology | Diarrhea patients | 2001-2002 | ^[56]^ |
|  | Chuzhou | 3 | Pathobiology | Case report | 1992-1993 | ^[55]^ |
|  |  | 1.42 (6/423) | Pathobiology | General population | 2001 | ^[50]^ |
|  | Liu'an | 1.34 (6/447) | Pathobiology | General population | 2001 | ^[50]^ |
|  | Ma'anshan | 1.02 (5/489) | Pathobiology | General population | 2014-2015 | ^[52]^ |
|  | Wuhu | 1092 (67/3498) | Pathobiology | Diarrhea patients | 1989 | ^[54]^ |
|  |  | 1.29 (6/464) | Pathobiology | General population | 2001 | ^[50]^ |
|  | Xuancheng | 9.36 (32/342) | Pathobiology | HIV/AIDS patients | 2010-2012 | ^[57]^ |
|  | Anqing | 1.21 (5/413) | Pathobiology | General population | 2001 | ^[50]^ |
|  |  | 1.81 (6/331) | Pathobiology | General population | 2014-2015 | ^[52]^ |
|  | Huangshan | 1.40 (7/500) | Pathobiology | General population | 2001 | ^[50]^ |
| Fujian Province | Fuzhou | 9.94 (16/161) | Pathobiology | Diarrhea patients | 1989 | ^[58]^ |
|  |  | 0.64 (20/3116) | Pathobiology | Diarrhea patients | 1996-2001 | ^[59]^ |
|  | Xiamen | 2 | Pathobiology | Case report | 1992 | ^[60]^ |
|  | Zhangzhou | 3.81 (8/210) | Pathobiology | Diarrhea patients | 1990 | ^[61]^ |
|  |  | 2.06 (4/191) | Pathobiology | Diarrhea patients | 1990-1992 | ^[62]^ |
|  |  | 2.82 (7/248) | Pathobiology | Diarrhea patients | Before 2004 | ^[63]^ |
|  | Nanping | 0.23 (1/413) | Pathobiology | General population | 1990 | ^[64]^ |
|  |  | 8.05 (31/385） | Pathobiology | Diarrhea patients | 1991 | ^[65]^ |
|  | Ningde | 1.67 (6/359) | Pathobiology | General population | 1991 | ^[66]^ |
| Jiangxi Province | Ganzhou | 1.65 (2/121) | Pathobiology | Diarrhea patients | 1989 | ^[67]^ |
|  |  | 1.90 (4/210) | Pathobiology | Diarrhea patients | Before 1991 | ^[68]^ |
|  | Shangrao | 70.20 (688/980) | Immunology | General population | 2005 | ^[49]^ |
| Shandong Province | Jinan | 0.90 (1/111) | Pathobiology | Diarrhea patients | 1989-1990 | ^[69]^ |
|  |  | 66.67 (72/108) | Pathobiology | Malignant tumor patients | Before 2007 | ^[70]^ |
|  | Qingdao | 2.38 (87/3655) | Pathobiology | General population | 1989-1996 | ^[71]^ |
|  | Weifang | 2.01 (75/3729) | Pathobiology | General population | 1996-1998 | ^[72]^ |
|  | Jining | 0.78 (1/129) | Pathobiology | Diarrhea patients | 1989-1990 | ^[69]^ |
|  | Tai'an | 0.38 (1/264) | Pathobiology | Diarrhea patients | 1989-1990 | ^[69]^ |
|  | Binzhou | 6.25 (7/112) | Immunology | Diarrhea patients | Before 2009 | ^[73]^ |
|  | Heze | 2.53 (6/237) | Pathobiology | Diarrhea patients | 2002-2003 | ^[74]^ |
| Henan Province | Zhengzhou | 0.11 (2/1753) | Molecular biology | Diarrhea patients | 2005-2006 | ^[75]^ |
|  |  | 4 | Molecular biology | Case study | 2007-2008 | ^[76]^ |
|  |  | 0.10 (2/1996) | Pathobiology | Diarrhea patients | 2009-2010 | ^[77]^ |
|  |  | 2 | Molecular biology | Case study | Before 2011 | ^[78]^ |
|  |  | 2.00 (2/100) | Pathobiology | Diarrhea patients | 2015 | ^[79]^ |
|  | Kaifeng | 2.48 (12/483) | Pathobiology | Diarrhea patients | 1990 | ^[80]^ |
|  |  | 0.22 (2/912) | Pathobiology | General population | 1995-1996 | ^[81]^ |
|  |  | 0.19 (6/3130) | Pathobiology | General population | 1995-1998 | ^[82]^ |
|  |  | 4 | Molecular biology | Case study | 2007-2008 | ^[76]^ |
|  |  | 0.10(6/6093) | Pathobiology | Diarrhea patients | Before 2009 | ^[83]^ |
|  | Anyang | 4 | Molecular biology | Case study | 2007-2008 | ^[76]^ |
|  |  | 0.15 (3/1949) | Pathobiology | General population | 2007-2008 | ^[84]^ |
|  | Jiaozuo | 0.57 (4/699) | Pathobiology | Diarrhea patients | 1991 | ^[85]^ |
|  | Xuchang | 8.00 (20/250) | Pathobiology | Diarrhea patients | 1995-1996 | ^[86]^ |
|  | Nanyang | 5 | Molecular biology | Case study | 2006-2007 | ^[87]^ |
|  | Zhoukou | 1 | Pathobiology | Case report | 1991 | ^[88]^ |
|  |  | 1.12 (12/1074) | Pathobiology | Diarrhea patients | 1991 | ^[89]^ |
|  | Zhumadian | 16.11 (24/149) | Pathobiology | HIV/AIDS patients with diarrhea | 2010 | ^[90]^ |
| Hubei Province | Wuhan | 3.02 (9/298) | Molecular biology | Diarrhea patients | 2014-2015 | ^[91]^ |
|  |  | 2.00 (10/500) | Molecular biology | Diarrhea patients | 2016 | ^[92]^ |
|  | Shiyan | 6.59 (62/941) | Pathobiology | General population | Before 2007 | ^[93]^ |
|  |  | 4.67 (119/2549) | Pathobiology | General population | Before 2009 | ^[94]^ |
|  |  | 48.85 (106/217) | Pathobiology | Malignant tumor patients | Before 2010 | ^[95]^ |
|  | Jingzhou | 3.70 (8/216) | Molecular biology | General population | 2017 | ^[96]^ |
|  | Xianning | 5.60 (58/1035) | Pathobiology | General population | 2012 | ^[97]^ |
|  |  | 3.19 (36/1128) | Pathobiology | General population | Before 2013 | ^[98]^ |
| Hunan Province | Changsha | 4.90 (5/102) | Pathobiology | Diarrhea patients | 1997 | ^[99]^ |
|  |  | 3.85 (2/52) | Pathobiology | Diarrhea patients | 1997 | ^[100]^ |
|  |  | 9.18 (27/294) | Pathobiology | Drug addict | 2000 | ^[101]^ |
|  |  | 19.05 (172/903) | Pathobiology | Drug addict | 2002 | ^[102]^ |
|  |  | 19.05 (34/179) | Pathobiology | Drug addict | 2002 | ^[103]^ |
|  |  | 0.84 (3/356) | Pathobiology | Tuberculosis patients | 2000-2002 | ^[104]^ |
|  |  | 3.33 (16/480) | Molecular biology | Patients with hepatitis B virus-associated acute-on-chronic liver failure | 2002-2005 | ^[105]^ |
|  | Hengyang | 0.94 (7/747) | Pathobiology | General population | 1990-1991 | ^[106]^ |
|  |  | 44.09 (82/186) | Pathobiology | HIV/AIDS patients with diarrhea | 2007-2009 | ^[107]^ |
|  |  | 13.16 (20/152) | Pathobiology | HIV/AIDS patients | 2014 | ^[108]^ |
|  | Zhuzhou | 1.16 (5/430) | Pathobiology | General population | 1990-1991 | ^[106]^ |
|  | Chenzhou | 2.22 (57/2562) | Pathobiology | General population | 1990-1991 | ^[106]^ |
| Guangdong Province | Guangzhou | 3 | Pathobiology | Case report | 1989 | ^[109]^ |
|  |  | 1.47 (4/271) | Pathobiology | Diarrhea patients | 1990 | ^[110]^ |
|  |  | 2.75 (5/182) | Pathobiology | General population | Before 1992 | ^[111]^ |
|  |  | 14.29 (2/14) | Pathobiology | HIV/AIDS patients | Before 2008 | ^[112]^ |
|  |  | 3.45 (12/348) | Molecular biology | Diarrhea patients | 2012-2013 | ^[113]^ |
|  |  | 17.33 (78/450) | Immunology | HIV/AIDS patients | 2013 | ^[114]^ |
|  |  | 15.00 (15/100) | Pathobiology | HIV/AIDS patients | 2015-2018 | ^[115]^ |
|  | Shenzhen | 2.68 (3/112) | Pathobiology | HIV/AIDS patients | Before 2008 | ^[112]^ |
|  | Jieyang | 1 | Pathobiology | Case report | 1991 | ^[116]^ |
|  |  | 2.31 (16/693) | Pathobiology | Diarrhea patients | 1990-2000 | ^[117]^ |
|  | Donguan | 3.42 (19/556) | Pathobiology | Chronic hepatitis B patients | 2004-2005 | ^[118]^ |
| Guangxi Zhuang  Autonomous Region | Nanning | 0.50 (2/400) | Molecular biology | General population | 2016 | ^[119]^ |
|  | Liuzhou | 1.37 (1/73) | Pathobiology | Diarrhea patients | 1992 | ^[120]^ |
|  | Guilin | 1.85 (1/54) | Pathobiology | Diarrhea patients | 1991-1992 | ^[121]^ |
|  |  | 0.46 (2/435) | Molecular biology | HIV/AIDS patients | 2013-2014 | ^[122]^ |
|  |  | 16.00 (4/25) | Pathobiology | General population | 2015 | ^[123]^ |
|  |  | 4.00 (12/298) | Immunology | General population | 2017 | ^[124]^ |
|  | Hechi | 2.33 (6/258) | Molecular biology | HIV/AIDS patients | 2014-2015 | ^[125]^ |
| Hainan Province | Haikou | 2 | Pathobiology | Case report | 1995 | ^[126]^ |
| Chongqing City |  | 8.00 (20/250) | Pathobiology | Diarrhea patients | 1985 | ^[127]^ |
|  |  | 4.09 (34/830) | Pathobiology | Diarrhea patients | 1989-1991 | ^[128]^ |
|  |  | 1 | Molecular biology | Case study | Before 1996 | ^[129]^ |
|  |  | 3.93 (42/1238) | Pathobiology | General population | 2011 | ^[130]^ |
| Sichuan Province | Chengdu | 2.96 (12/406) | Pathobiology | Diarrhea patients | 1998-2000 | ^[131]^ |
|  | Guangyuan | 0.55 (1/180) | Pathobiology | Diarrhea patients | 2015-2017 | ^[132]^ |
|  | Ya'an | 16.64 (13/79) | Pathobiology | HIV/AIDS patients | 2008-2013 | ^[133]^ |
|  | Liangshan | 12.03 (74/615) | Pathobiology | General population | 2014 | ^[134]^ |
| Guizhou Province | Guiyang | 0.67 (1/150) | Pathobiology | Diarrhea patients | 1990-1997 | ^[135]^ |
|  | Qiannan | 2.11 (41/1946) | Pathobiology | General population | 1997 | ^[136]^ |
|  | Qianxinan | 1.15 (4/348) | Pathobiology | General population | 1997 | ^[136]^ |
| Yunnan Province | Kunming | 12.3 (9/73) | Pathobiology | Diarrhea patients | 1988 | ^[137]^ |
|  |  | 12.96 (7/54) | Pathobiology | Diarrhea patients | 1997-2000 | ^[138]^ |
|  |  | 4.65 (4/86) | Pathobiology | HIV/AIDS patients | Before 2008 | ^[112]^ |
|  |  | 60.00 (18/30) | Pathobiology | HIV/AIDS patients | 2009 | ^[139]^ |
|  |  | 0.1 (1/850) | Molecular biology | Diarrhea patients | 2014-2015 | ^[140]^ |
|  | Qujing | 2.3 (2/87) | Pathobiology | General population | 1987-1990 | ^[141]^ |
|  |  | 6.75 (6/89) | Pathobiology | Diarrhea patients | 1997-2000 | ^[138]^ |
|  | Yuxi | 2 | Pathobiology | Case report | 1990 | ^[142]^ |
|  |  | 1.35 (33/2450) | Pathobiology | General population | 1991 | ^[143]^ |
|  | Baoshan | 0.62 (2/324) | Molecular biology | HIV/AIDS patients | 2017 | ^[144]^ |
|  | Honghe | 3.90 (3/77) | Pathobiology | Diarrhea patients | 1997-2000 | ^[138]^ |
|  |  | 0.57 (3/524) | Pathobiology | General population | 2000 | ^[145]^ |
|  | Pu'er | 2.17 (1/46) | Pathobiology | Diarrhea patients | 1997-2000 | ^[138]^ |
|  | Xishuangbanna | 3.18 (2/63) | Pathobiology | Diarrhea patients | 1997-2000 | ^[138]^ |
|  | Dali | 16.80 (84/500) | Pathobiology | Drug addict | 2002-2004 | ^[146]^ |
|  | Nujiang | 3.13 (1/32) | Pathobiology | Diarrhea patients | 1997-2000 | ^[138]^ |
| Shaanxi Province | Xi'an | 1.58 (3/190) | Pathobiology | Diarrhea patients | 1990 | ^[147]^ |
|  |  | 1 | Pathobiology | Case report | 1991 | ^[148]^ |
|  |  | 7.79 (36/462) | Pathobiology | Diarrhea patients | 1997-1998 | ^[149]^ |
| Gansu Province | Lanzhou | 7.00 (13/186) | Pathobiology | Diarrhea patients | 1997-1998 | ^[150]^ |
|  |  | 19.13 (22/115) | Pathobiology | Diarrhea patients | 1997-1998 | ^[151]^ |
|  |  | 9.31 (54/580) | Pathobiology | Diarrhea patients | 1998-2003 | ^[152]^ |
|  |  | 4.17 (25/600) | Pathobiology | Diarrhea patients | 2010 | ^[153]^ |
|  | Jiuquan | 2.77 (8/288) | Pathobiology | Diarrhea patients | 1997-1998 | ^[151]^ |
|  | Zhangye | 2.52 (11/437) | Pathobiology | Diarrhea patients | 1997-1998 | ^[151]^ |
|  | Longnan | 1 | Pathobiology | Case report | 1993 | ^[154]^ |
| Qinghai Province | Xining | 1 | Pathobiology | Case report | Before 2009 | ^[155]^ |
|  |  | 1.00 (1/100) | Pathobiology | Diarrhea patients | 2016-2018 | ^[156]^ |
| Xinjiang Uygur Autonomous Region | Urumqi | 10.8 (4/37) | Pathobiology | HIV/AIDS patients | 2009-2011 | ^[3]^ |
|  |  | 16.5 (38/230) | Molecular biology | Diarrhea patients | Before 2017 | ^[157]^ |
|  | Ili | 1.8 (2/114) | Pathobiology | General population | 1990-1992 | ^[62]^ |
|  | Turpan | 21.57(14/65) | Pathobiology | HIV/AIDS patients with diarrhea | 2012-2016 | ^[158]^ |
|  | Bayinggol | 4.7(53/1124) | Pathobiology | General population | 1990-1992 | ^[159]^ |
| Taiwan Province | Taipei | 0.7(2/300) | Pathobiology | Diarrhea patients | 1988 | ^[160]^ |
|  |  | 1.2(4/332) | Molecular biology | HIV/AIDS patients | 2001-2003 | ^[161]^ |
|  | New Taipei | 0.5(1/217) | Molecular biology | Diarrhea patients | 2015-2016 | ^[162]^ |
|  | Changhua | 1 | Molecular biology | Case study | Before 2011 | ^[163]^ |

**References**

1. Lu SQ, Wang F, Zhang K, Xu L. Study on genetic approach in the detection of *Cryptosporidium parvum* and *Giardia lamblia* in acquired immunodeficiency syndrom patients. Chin J Epidemiol. 2006;27(10):884-8. (in Chinese)
2. Wang HF, Liu J, Yu JF, Sun F. A Rapid Immunoassay for detection of *Cryptosporidium parvum* and *Giardia lamblia* antigens in human stool and its application. J Mod Lab Med. 2010;25(4):65-6. (in Chinese)
3. Zhou YL, Wang YG, Li P, Liu JY, Wang HZ. Clinical study on 311 patients with AIDS related chronic diarrhea. Chin J Exp Clin Infect Dis. 2012;6(3):191-4. (in Chinese)
4. Wang HZ, Guo JJ, Li M, Li RH, Zhang Y, Lu Y, et al. Study of *Cryptosporidium* infection in HIV/AIDS patients with chronic diarrhea. Int J Lab Med. 2015;36(22):3300-1. (in Chinese)
5. Feng W, Gu X, Sui W, Zhang M, Lu B, Wang M, et al. The application and epidemiological research of xTAG GPP multiplex PCR in the diagnosis of infectious diarrhea. Natl Med J China. 2015;95(6):435-9. (in Chinese)
6. Zhan SZ, Wang AD, Ma J, Wang ZL, Geng GY. Investigation on human cryptosporidiosis in urban and suburban counties of Tianjin (report of three cases). Tianjin Med J. 1992;8:491-2. (in Chinese)
7. Zhang YR, Ji WH, Liu X, Yu AD, Zhu JR. Investigation of cryptosporidiosis in children in Tianjin. Chin J Parasitic Dis Control. 1993;4:317. (in Chinese)
8. Zhan W, Chen YG, Jing LY, He SH, Liang ZH. Investigation of *cryptosporidium* infection rate among diarrheapatients in four cities in China. Chin J Dis Control Prev. 2002;6(1):76-7. (in Chinese)
9. Peng MM, Matos O, Gatei W, Das P, Stantic-Pavlinic M, Bern C, et al. A comparison of *Cryptosporidium* subgenotypes from several geographic regions. J Eukaryot Microbiol. 2001;Suppl:28S-31S.
10. Wu HB, Zhang Y, Li GM, Zhu Z. 422 cases of children with acute diarrhea in Peking University Binhai Hospital were tested for pathogenic microorganisms In 2014. Mat Child Health Care Chin. 2015;30:4314-6. (in Chinese)
11. Wei YJ. Analysis of the detection results of pathogenic microorganisms in infants with acute diarrhea. Guide Chin Med. 2014;12(10):127-8. (in Chinese)
12. Miao ZF, Lv JP, Zhu X, Zhang TS, Shan LJ, Guo JS, et al. First report of infantile cryptosporidiosis in Hebei Province. Chin J Pest Control. 1994;3:210–1. (in Chinese)
13. Miao ZF, Zhu X, Lv JP, Zhang WF, Zhang TS. Study on infantile infection with cryptosporidiosis. Chin J Pest Control. 1994;3:205–8. (in Chinese)
14. Chen SL, Cao ZR, Li HL, Geng SJ. *Cryptosporidium* infection in diarrheal patients and mini-review. J Hebei Med Coll Contin Educ. 1996;3:18-9. (in Chinese)
15. Zhou YC, Chen SL, Cao ZR. *Cryptosporidium* infection in a patient with chronic diarrhea and detection of immune status. Lif Inf Pre Med. 1999;5(3):284. (in Chinese)
16. Li ZC, Li JM. Analysis of the test results of common pathogenic microorganisms causing acute diarrhea in infants and young children aged 0-5. Med Imaging Clin Exam. 2020;47:251. (in Chinese)
17. Guan JS, Cui SL, Yan XL, Zhang JZ. Investigation of *Cryptosporidium* enteritis in Hohhot area. Acta Acad Med Nei Mongol. 1989;11(1):47-8. (in Chinese)
18. Xie YT, Wang L, Cui XC, Jin GC, Tong YF, Tian XH, et al. *Cryptosporidium* infection of children in pastoral areas of Inner Mongolian. J Envir Hyg. 2015;5(6):504. (in Chinese)
19. Li Y, Bing YY, Cong F, An CL. Investigation of *Cryptosporidium* infection in diarrheal patients and drug users in Shenyang area. Chin J Zoonoses. 2006;22(5):473-5. (in Chinese)
20. Song JP, Zhao JX, Gao H, Liu Y, Yue HX, Zhang J, et al. Serological detection of *Cryptosporidium spp.* infection in outpatients in Changchun. Chin J Parasitol Parasitic Dis. 2011;29(3):239-41. (in Chinese)
21. Guo J. Analysis of pathogen detection of stool samples of HIV infected patients complicated with chronic diarrhea. Chin Contin Med Educ. 2017;9(13):63-4. (in Chinese)
22. Zhao XN, Zhang DM, Zhang LX, Liu XM. Investigation of *Cryptosporidium* infection in infants in Harbin area. Chin J Parasitic Dis Control. 1997;10(3):75. (in Chinese)
23. Niu Y, Li YH, Yu XH. Preliminary investigation of Cryptosporidium infection in diarrheal patients in Qiqihar area. J Qiqihar Med Coll. 2006, 27(2):184-5. (in Chinese)
24. Liu HX. Analysis of pathogenic microorganisms in fecal specimens of HIV patients with chronic diarrhea. J Practical Gynecol Endocrinol. 2017,4(15):64-6. (in Chinese)
25. Zhou HF, Zhu M, Yuan JL, Xu F, Chen YH, Zhang ZX, et al. A survey on *Cryptosporidium parvum* cryptosporidiosis in different people of Luwan district Shanghai. Shanghai J Prev Med. 2005;17(9):430-2. (in Chinese)
26. Feng Y, Wang L, Duan L, Gomez–Puerta LA, Zhang L, Zhao X, et al. Extended outbreak of cryptosporidiosis in a pediatric hospital, China. Emerg Infect Dis. 2012;18(2):312–4.
27. Qian FX, Shi M, Shen SL, Liu YH, Gao T. Investigation of *Cryptosporidium* infection in diarrheal adults with or without HIV/AIDS. Chin J Misdiagn. 2011,11(3):752. (in Chinese)
28. Chen S, Ai L, Tian L, Zhang Y, Tong X, Li H, et al. Investigation and fecal specimen detection of cryptozoite and other protozoon infection from patients with diarrhea. Chin J Zoonoses. 2012;28(8):815-9. (in Chinese)
29. Chen SH, Zhang YN, Li H, Cai YC, Chen JX. Analysis on Parasitic Infection of Clinical Samples from Hospitals in Shanghai during 2011-2013. Chin J Parasitol Parasit Dis. 2014;32(6):446-51. (in Chinese)
30. Jiang Y, Yuan Z, Liu H, Yin J, Qin Y, Jiang X, et al. Intestinal Protozoan Infections in Patients with Diarrhea - Shanghai Municipality, Zhenjiang City, and Danyang City, China, 2011-2015 and 2019-2021. China CDC Wkly. 2022;4(8):143-7.
31. Liu H, Shen Y, Yin J, Yuan Z, Jiang Y, Xu Y, et al. Prevalence and genetic characterization of *Cryptosporidium*, Enterocytozoon, Giardia and Cyclospora in diarrheal outpatients in China. BMC Infect Dis. 2014;14:25.
32. Zhang XP, He YY, Wang ZY, Zhang YG, Zhu Q, Jiang SF, et al. Investigation on *Cryptosporidium* infections in diarrhea patients from the general hospitals in Shanghai. Chin Trop Med. 2016;16(12):1183–6. (in Chinese)
33. Wang ZY, Zhang YG, Ma XJ, Jiang L, He YY, Zhu Q, et al. Investigation on common infection of diarrhea-causing parasitic protozoa in Shanghai during 2015-2019. Chin J Parasitol Parasit Dis. 2021;39(6):759-63. (in Chinese)
34. Han F, Wang L, Wang RZ, Ge JJ, Shen JP. Investigation of cryptopsoridiosis in humans in Nanjing, China. Chin J Zoonoses. 1989;5(5):51. (in Chinese)
35. Ge JJ, Shen JP, Hu BY. Laboratory diagnosis of enteritis caused by *Cryptosporidium*. Lab Med. 1990;5(3):65-6. (in Chinese)
36. Shen JP, Ge JJ. Investigation and analysis of the infection rate of *Cryptosporidium* among 2018 children in kindergartens. J Pract Pediatr. 1991;6(3):132. (in Chinese)
37. Tian SL, Zhang KY. An epidemiological survey of Cryptosporidium in children and a mini-review on research situation of human cryptosporidiosis in China. Chin J Public Health. 1991;7(4):169-71. (in Chinese)
38. Chen YG, Yao FB, Li HS, Shi WS, Dai MX, Lu M. *Cryptosporidium* infection and diarrhea in rural and urban areas of Jiangsu, People's Republic of China. J Clin Microbiol. 1992;30(2):492-4.
39. Du XL, Ge JJ, Qin YF, Meng R, Liu Y, Chu K, et al. The epidemiological study on cryptosporidiosis in outpatients of Nanjing children's hospital. J Tro Medicine. 2009;9(4):382-5. (in Chinese)
40. Yang PC, Zhang HY, Zhou W, Yin WG, Wu P, Zhang Ke, et al. *Cryptosporidium* infection in Nanjing City from 2015 to 2016. Chin J Schisto Control. 2017;29(6):752-5. (in Chinese)
41. Zhang LJ. Analysis of Pathogenic Microbiological Test on Infantile Acute Diarrhea. Chin Contin Med Educ. 2017;9(4):84-6. (in Chinese)
42. Chen YG, Dai MX, Yao FB. Investigation on *Cryptosporidium* infection in villagers of zhoupeng village, in Tongshan County. Acta Acad Med Xuzhou. 1990;1:40–2. (in Chinese)
43. Yao FB, Chen YG. Brief report on the therapeutic effect of allicin on infant cryptosporidiosis. Acta Acad Med Xuzhou. 1989;1:56-7. (in Chinese)
44. Zheng H, He J, Wang L, Zhang R, Ding Z, Hu W. Risk Factors and Spatial Clusters of *Cryptosporidium* Infection among School-Age Children in a Rural Region of Eastern China. Int J Environ Res Public Health. 2018 May 6;15(5):924.
45. Sun X, Gu GM, Gu GS, Pan YX, Cao MX, Tang JL. Investigation of cryptosporidiosis in patients with diarrhea in Haian County. Chin J Public Health. 1992;8(8):350. (in Chinese)
46. Lu SH ,Lin AF ,Chen R ,Wen LY, Cheng YZ, Chen XJ, et al. The diagnosis and analysis of *Cryptosporidium* infection in children of Zhejiang Province. Chin J Zoonoses. 2000;1:42-4. (in Chinese)
47. Xing WL, Yang L, Liang SH, Liu QZ, Zheng XY. Investigation of *Cryptosporidium* infection in diarrheal patients in Wenzhou. J Wenzhou Med Coll. 1999;2:29-30. (in Chinese)
48. Li C, Li XY, Fang ZX. A case of intracranial infection of *Cryptosporidium*. Chin J Zoonoses. 2003;19(1):131-2. (in Chinese)
49. Study on Sero-epidemiology of cryptosporidiosis in normal population of Chinese rural communities using recombinant *Cryptosporidium* antigen. Najing: Nanjing Medical University. 2007. (in Chinese)
50. Wang KX, Li CP, Wang J, Pan BR. Epidemiological survey of cryptosporidiosis in Anhui Province China. World J Gastroenterol. 2002;8(2):371-4.
51. Zu SX, Du MW. The discovery of human cryptosporidiosis in China (brief report). Acta Univ Med Anhui. 1987;22(4):246. (in Chinese)
52. Wang QQ, Guo JD, Cao ZG, Wang QZ, Liu DH, Wang TP. Investigation on human *Cryptosporidium* infection in local area of Anhui. Chin J Schisto Control. 2015;27(3):263-7. (in Chinese)
53. Tian LG, Wang TP, Cheng GJ, Wang FF, Tong XM, Guo J, et al. Cross–section study on co–infection of HIV and *Cryptosporidium*. Chin J Schisto Control. 2012;24(1):54-7. (in Chinese)
54. Cai R, Li CP, Wang J, Xu LF, He Y. An epidemiology survey of cryptosporidiosis with diarrhea in Huainan area. J Trop Dis Parasitol. 2003;1(1):26-8. (in Chinese)
55. Cheng TY, Zhang CZ. A report of 3 cases of infant cryptosporidiosis in Chuzhou City, Anhui Province. J Pract Parasit Dis. 1995;3(1):9. (in Chinese)
56. Zhu FC, Wen WM. Cryptosporidiosis in children in Wuhu. Chin J Epidemiol. 1991;12(5):286-8. (in Chinese)
57. Liu DH, Wang TP, Li YF, Yang WP, Guo JD. Co–infection status of HIV and Cryptosporidium in Xuancheng City. Parasitoses Infect Dis. 2013;11(4):197-200. (in Chinese)
58. Su QP, Chen DG, Hua XL, Chen S, Zhao ZQ, Huang MH, et al. Cryptosporidiosis from infants in Fuzhou region. Chin J Zoonoses. 1989;5:35-6. (in Chinese)
59. Chen H, Li CH, Xie ML, Wei XY, Yao LJ. The relationship between diarrhea and the infection of intestinal protozoa in Fuzhou district. Strait J Prev Med. 2002(6):14-6. (in Chinese)
60. Ye JY, Chen WH, Guo MX, Guo LC. A report of 2 cases of cryptosporidiosis in humans (infants and young children) found for the first time in Xiamen area. Chin J Zoonoses. 1993;9(5):60-1. (in Chinese)
61. Su QP, Huang MH, Gao QF, Zhang JL, Wang BS, Zhang YS, et al. The first report of human cryptosporidiosis in Zhangzhou City of Fujian Province. Chin J Parasitol Parasitic Dis. 1994;12(1): 79-80. (in Chinese)
62. Hong LX, Yang WC, Peng WF, Cui HJ. Survey on the *Cryptosporidium* and intestinal protozoa of man and animal. J Xiamen Univ. 1996;2:305-8. (in Chinese)
63. Xu HZ, Lin GH, Meng JF, Huang ZM. Investigation of *Cryptosporidium* infection in diarrheal patients in Longhai City of Fujian Province. Chin J Zoonoses. 2005;21(4):282. (in Chinese)
64. Su QP, Zheng CR, Zhou BX, Chen HC, Xiao ZL, Shen QY, et al. Investigation on *Cryptosporidium* Infection in Children and Dairy Cows in Shunchang County. Chin J Zoonoses. 1991;7(3):18-9. (in Chinese)
65. Zheng CR, Xiao ZL. Epidemiological investigation of Cryptosporidium infection and cryptosporidiosis in infants in Nanping of Fujian Province. Chin J Parasitic Dis Control. 1993;6(1):64. (in Chinese)
66. Xie WQ, Zheng ZZ, Zhao Y, Zhuo BY, Xiao YL. The first report of six cases of *Cryptosporidium* infection in Xiapu County in Fujian Province. Chin J Zoonoses. 1992;8(3):23–6. (in Chinese)
67. Su SL, Chen GF, Wu GH, Huang AM. Investigation of cryptosporidiosis in children with diarrhea in Ganzhou City. J Gannan Med Coll. 1992;12(1):52. (in Chinese)
68. Xie ZJ, Zhang RQ, Huang WF, Liao YG, Su SL. Epidemiological and clinical study on intestinal parasites in adults with chronic diarrhea in Ganzhou area. J South Med Univ. 2008;28(6):1035-6. (in Chinese)
69. Chen XC, Su LY, Sai SY, Liu JH, Yang GL, Song JM. Investigation of human *Cryptosporidium* in south and central Shandong Province. J Taishan Med Coll. 1991;4:359-62. (in Chinese)
70. Xin L, Cui W, Liang R, Ji R, Sun X, Li R. Investigation on the infection of *Cryptosporidium* among malignant tumor patients. J Pathog Biol. 2007;2(4):307-8. (in Chinese)
71. Gong YX, Cao SQ, Shi XZ, Han M, Zhou SC, Hao JH. Survey of human *Cryptosporidium* infection in Qingdao area. Acta Acad Med Qingdao. 1997;2:28-9. (in Chinese)
72. Cui W, Liang RW, Wang ZZ. Epidemiological investigation of *Cryptosporidium* infection in children in Weifang area. Chin J Prev Med. 2001;35(4):73. (in Chinese)
73. Zhang Z, Li HX, Liu CH, Niu Q, Bai XL. An epidemiological survey of *Crptosporidium* in diarrheal patients as well as application of a recombinant cp23antigen in the detection of C. parvum in fecal specimens. Shandong Med J. 2009. 49(11):52-3. (in Chinese)
74. Zhou YX. Investigation of *Cryptosporidium* infection in diarrheal patients in Heze City during the period of 2002-2003. Endemic Dis Bull. 2006;21(2):38. (in Chinese)
75. Zhu HL. Research on *Cryptosporidium* species/genotype/subgenotype of Henan Strain in Zhengzhou. Zhengzhou: Henan Agricultural University. 2007. (in Chinese)
76. Wang R, Zhang X, Zhu H, Zhang L, Feng Y, Jian F, et al. Genetic characterizations of *Cryptosporidium spp*. and *Giardia duodenalis* in humans in Henan, China. Exp Parasitol. 2011;127(1):42-5.
77. Xu LN, Wu GQ, Xi JW, Qi M, Yang N, Zhang LX, et al. Prevalence of intestinal parasitic infection in children in a hospitalized children in Zhengzhou. J Trop Med. 2011;11(1):178. (in Chinese)
78. Zhu H, Zhao J, Wang R, Zhang L. Molecular identification of a rare subtype of *Cryptosporidium hominis* in infants in China. PLoS One. 2012;7(8):e43682.
79. Tang Q. Analysis of test results of common pathogenic microorganisms for acute diarrhea in children of different genders and ages. J Med Theor Prac. 2019;32(8):1236-7. (in Chinese)
80. Su YP, He LJ, Song JD, Lu LF, Zhang KR, Zhang QF. Investigation of *Cryptosporidium* infection in diarrhea infants in Henan Province. Henan J Prev Med. 1991;2(1):533-5. (in Chinese)
81. Lu JZ, Su YP, He LJ, Li JA, Li XW, Ren YM. Three pediatric cases cryptosporidiosis in Wuzhi County of Henan Province. Chin J Parasitol Parasitic Dis. 1994;2:72. (in Chinese)
82. Li W, He LJ, Yan QY, Su YP, Zhao KY. Investigation on cryptosporidiosis in Henan. Strait J Prev Med. 2000;5:4-5. (in Chinese)
83. Wang Q, Zhou Y, Lv B, Fu KD, Chen L, Wang YQ, et al. Prevalence of intestinal parasite infection in Kaifeng City, Henan Province. J Trop Med. 2009;9(5):510-4. (in Chinese)
84. Wang RJ, Qi M, Zhao YF, Zhang XS, Ning CS, Zhang LX. Prevalence of Intestinal Parasitic Infection in Children in Linzhou, Henan Province. J Trop Med. 2009;9(10):1184-7. (in Chinese)
85. Lu JZ, Su YP, He LJ, Li JA, Li XW, Ren YM. Three pediatric cases cryptosporidiosis in Wuzhi County of Henan Province. Chin J Parasitol Parasitic Dis. 1994;2:72. (in Chinese)
86. Zhang YY, Yu FY, Zhang XF, Zhang HY, An YQ. Investigation and clinical analysis of *Cryptosporidium* infection in children with diarrhea. Chin J Appl Clin Pediatr. 1997;12(2):127. (in Chinese)
87. Wang T, Wang AH. Discussion on the etiological diagnosis method of AIDS complicated with *Cryptosporidium* infectious diarrhea. Tianjin Med J. 2008;36(8):622-3. (in Chinese)
88. Li GD, Zhao YM, Li GZ, Wang LM, Wu XY, Wang L. A case report of cured severe cryptosporidiosis. Chin J Zoonoses. 1993;9(2):48-50. (in Chinese)
89. Su YP, Liu H, Yan XX, Sun QL, He ZL, Li GD, et al. Finding of cryptosporidiosis in humans in Zhoukou area of Henan Province. J Pract Parasitic Dis. 1994;2(3):41. (in Chinese)
90. Wang HZ, Jiao BX, Tian JH, Li M, Guo J, Liu Y, et al. Detection of *Cryptosporidium* infection among HIV/AIDS patients with chronic diarrhea in Beijing, Henan and Xinjiang of China. Chin J Epidemiol. 2011;32(9):927-9. (in Chinese)
91. Liu X, Mao T, Wu P, Zhou R. Molecular epidemiology of *Cryptosporidium* infection in infants with diarrhea in Wuhan City. Chin J Schisto Control. 2017;29(2):188-91. (in Chinese)
92. Wang T, Fan Y, Koehler AV, Ma G, Li T, Hu M, et al. First survey of *Cryptosporidium*, *Giardia* and *Enterocytozoon* in diarrhoeic children from Wuhan, China. Infect Genet Evol. 2017;51:127-31.
93. Zhu MS, Song MH. *Cryptosporidium* infection in students of some primary and middle school in Shiyan City of Hubei Province. Chin J School Health. 2007;28(6):549. (in Chinese)
94. Zhu MS, Zhu J, Wang SJ, Song MH. A survey of *Cryptosporidium* infection among humans being in Shiyan, China. J Pathog Biol. 2009;4(9):685-6. (in Chinese)
95. Zhu J, Zhu MS, Wang SJ, Song MH. Investigation on the infection of *Cryptosporidium* among malignant tumor patients in Shiyan. J Trop Med. 2010,10(5):553-4. (in Chinese)
96. Wan LX, He ZA, Wang L, Zhang R. *Cryptosporidium* infection in children swimming in public swimming pools in Jingzhou, Hubei province. J Environ Health. 2018;35(1):69-71. (in Chinese)
97. Min HL, Chen SM, Hu JX. Investigation on *Cryptosporidium* infection in children in Xianning area. J Public Health Prev Med. 2014;25(6):111-2. (in Chinese)
98. He ZA, Fu LX, Zhou WS, Chen J, Tian BQ, Wang L, et al. Investigation on *Cryptosporidium* infection of children in rural area of Chibi City, Hubei Province. J Public Health Prev Med. 2013;24(6):72-4. (in Chinese)
99. Huang MZ, Guan L, Zhou CX, Li DQ, Hu B. Infection of *Cryptosporidium* in child patients with diarhea in Changsha. Bull Hunan Med Univ. 1998;3:38-9. (in Chinese)
100. Huang MZ, Zhou CX, Guan L, Li DQ, Liu YY, Yu B, et al. Study of *Cryptosporidium* infection in adult patients with diarrhea. Curr Physician. 1998;3(10):42-3. (in Chinese)
101. Huang MZ, Guan L, Li DQ, Liu AZ, Dai WP, Chen X. Investigation of *Cryptosporidium* infection in male intravenous drug users. Chin J Zoonoses. 2002;18(2):131. (in Chinese)
102. Huang MZ, Guan L, Xie MZ, Li DQ, Zhou J, Li ZY, et al. Study on condition of *Cryptosporidium* infection among male drug users in detoxification institute in Changsha City. Chin J Public Health. 2003;19(3):49-51. (in Chinese)
103. Li DQ, Li HH, Huang MZ, Dai WP. Study on the incidence of *Cryptosporidium* infection and the state of immune function of intravenous drug abusers. Chin J Zoonoses. 2005;2:153-5. (in Chinese)
104. Zeng SH, Xiang YE, Yi SL. *Cryptosporidium* infection of human respiratory tract and exploration of examination methods. Pract Prev Med. 2003;10(1):103. (in Chinese)
105. Yu Z, Li F, Zeng Z, Huang Z, Fan Z, Jin Y, et al. Prevalence and clinical significance of *Cryptosporidium* infection in patients with hepatitis B virus–associated acute–on–chronic liver failure. Int J Infect Dis. 2011;15(12):e845-8.
106. Lu LA, Li CC, Fan ZZ, Chen YL. Fingding and epidemiological investigation of zoonotic cryptosporidiosis in humans and livestock in Hunan Province. Chin J Zoonoses. 1992;8(2):43-4. (in Chinese)
107. Tang XC, Liu P, Gao XJ. Examination of Cryptozoites in the AIDS Patients' Night Soil from Diarrhea. J Clin Res. 2010;27(5):847-8.
108. Xie RH, Chen GX, Ouyang SS. Analysis status of intestinal parasite infection among HIV/AIDS patients in Hengyang. Chin J Immun. 2015;31(5):695-7. (in Chinese)
109. Wang JQ, Qu XM. Three cases of cryptosporidiosis in a family. J New Med. 1991;22(8):435. (in Chinese)
110. Zheng HB, Wang Y, Yao YX. Clinical Features of 4 Cases of infant *Cryptosporidium* enteritis. Acad J Guangzhou Med Coll. 2000;28(4):44-6. (in Chinese)
111. Li WS, Shen SM. A preliminary report on the investigation of *Cryptosporidium* infection in humans and animals Guangdong Province. J First Mil Meal Univ. 1994;14(1):41. (in Chinese)
112. Le XH, Wang H, Gou JZ, Chen XC, Yang GL, Yang QT, et al. Detection of *Cryptosporidium* infection among AIDS patients in Guangdong and Yunnan. Chin J Exp Clin Virol. 2008;22(5):339-41. (in Chinese)
113. Yao Y, Chen H, Liu X, Xiao N, Xiao Y, Huang Y, et al. Molecular epidemiological studies of cryptosporidiosis diarrhea in children of Guangzhou sentinel hospital. J Tro Medicine. 2014;14(1):60-3. (in Chinese)
114. Pang XL, Chen SY, Gao K, Mai HX, Han ZG, Xu HF, et al. Serum epidemiological analysis of opportunistic infection of pathogenic protozoa in HIV/AIDS. J Trop Med. 2015;15(10):1425-8. (in Chinese)
115. Huang FH, Tian Q. Detection and analysis of pathogenic microorganisms in stool samples of 100 HIV-infected patients with chronic diarrhea. Home Med. 2018;11:147. (in Chinese)
116. Lu DF, Xing PH. The first case of infant cryptosporidiosis in Jieyang City. Guangdong Med J. 1994;1:64. (in Chinese)
117. Cai XS, Lu DF, Chen SQ, Chen YJ, Wang T, Zheng DC. Etiological and clinical study of protracted and chronic diarrhea in children. Guangdong Med J. 2002;23(1):47-9. (in Chinese)
118. Li FW, Zhang XH, Wu FQ, Chen WN. Investigation of *cryptosporidium* infection in patients with chronic hepatitis b. New Med. 2007;38(2):81-3. (in Chinese)
119. Xu N, Liu H, Jiang Y, Yin J, Yuan Z, Shen Y, et al. First report of *Cryptosporidium viatorum* and *Cryptosporidium occultus* in humans in China, and of the unique novel *C. viatorum* subtype XVaA3h. BMC Infect Dis. 2020;20(1):16.
120. Gan ZG, Chen JM, He XC, Luo J, Li YS, Pan QH, et al. Epidemiological survey of *Cryptosporidium* in humans and livestock in Liuzhou City of Guangxi. Chin J Parasitic Dis Control. 1995;8(1):20. (in Chinese)
121. Jiang JX, Ou WL, Lao HB. A case report of infantile cryptosporidiosis. Chin J Parasitol Parasitic Dis. 1995;13(2):77. (in Chinese)
122. Wang ZF, Jiang ZH, Yu BX, Zhou DS, Lin Y, Tang WQ. Preliminary study on infection status and gene types of *Cryptosporidium* among HIV/AIDS patients in Guangxi. Chin J Schisto Control. 2016;28(5):550-3. (in Chinese)
123. Li J, Ren Y, Chen H, Huang W, Feng X, Hu W. Risk Evaluation of Pathogenic Intestinal Protozoa Infection Among Laboratory Macaques, Animal Facility Workers, and Nearby Villagers From One Health Perspective. Front Vet Sci. 202;8:696568.
124. Wan LX, Zhong GM, Huang CG, Lin Y, Shi ZH, Wang L, et al. Prevalence of *Cryptosporidium* infection in child swimmers in Guilin. Chin J Microbiol. 2018;30(7):818-20. (in Chinese)
125. Su H, Huang X, Qin Y, Qin S, Huang Z, Wu N, et al. An investigation on the gene types of *Cryptosporidium* among HIV/AIDS patients. J Med Pest Control. 2017;33(2):151-3. (in Chinese)
126. Lei Y, Han J, Ruan HQ, You J. A report of 2 cases of infant cryptosporidiosis in Hainan Province. Hainan Med J. 1997;2:138. (in Chinese)
127. Zhou YH. *Cryptosporidium* infection and enteritis in humans in Chongqing. J Chongqing Med Univ. 1987;1:3. Chinese.
128. Li WM, Cui FW, Zheng CZ, Yang Y. Clinical study on cryptosporidial enteritis and its treatment with Chinese herb medicines in children. Acta Acad Med Mil Tertiae. 1993;15(6):513-6. (in Chinese)
129. Ma L, Chen YT, Liu YH. Detection of cryptosporidium parvum in fecal samples by polymerase chain reaction. Chin J Parasitol Parasitic Dis. 1996;2:29-32. (in Chinese)
130. Zhang RP, Li XY, Li H, Pu CW, Feng L, Feng CY. *Cryptosporidium* in eastern Chongqing. Parasitoses Infect Dis. 2012;10(2):72-4. (in Chinese)
131. Zhang YJ, Luo P, Gao R. Investigation of *Cryptosporidium* infection in children with diarrhea in a children's hospital in Chengdu City. J Practical Parasitic Dis. 2001;9(2):72-3. (in Chinese)
132. Zhang H, He XW, Ren J. Analysis of common pathogenic microorganisms causing of different gender and age acute diarrhea in children. J Clin Exp Med. 2018;17(8):880-3. (in Chinese)
133. .Su YQ. Detection and analysis of pathogenic microorganisms in fecal specimens of patients with HIV infection complicated with chronic diarrhea. Chin Health Ind. 2014;11(6):24-5. (in Chinese)
134. Yang Y, Zhou YB, Xiao PL, Shi Y, Chen Y, Liang S, et al. Prevalence of and risk factors associated with *Cryptosporidium* infection in an underdeveloped rural community of southwest China. Infect Dis Poverty. 2017;6:2.
135. Chen Y, Lang SY, Li JH, Qiu XL. Investigation of opportunistic parasitic infections in some populations in Guiyang area. J Guiyang Med Coll. 1999;24(3):243-4. (in Chinese)
136. Rong JQ, Wu GP, Chen LH, Yu YS. Epidemiological survey of human cryptosporidiosis in Qiannan area. J Qiannan Med Coll National. 1999;1:52-4. (in Chinese)
137. Zuo YX, Chen FQ, Fang L. Finding of human and calf cryptosporidiosis in Yunnan Province and experimental infections. Chin J Zoonoses. 1990;3:34-6. (in Chinese)
138. Zhang BX, Yu H, Zhang LL, Tao H, Li YZ, Li Y, et al. Prevalence survey on *Cyclospora cayetanensis* and *Cryptosporidium ssp.* in diarrhea cases in Yunnan Province. Chin J Parasitol Parasitic Dis. 2002;20(2):106-8. (in Chinese)
139. Wang L, Pu D. Investigation of *Cryptosporidium* infection in AIDS patients in Kunming. J Pract Med Tech. 2011;18(4):360-1. (in Chinese)
140. Zhang SX, Zhou YM, Xu W, Tian LG, Chen JX, Chen SH, et al. Impact of co-infections with enteric pathogens on children suffering from acute diarrhea in southwest China. Infect Dis Poverty. 2016;5(1):64.
141. Wang YK, Yu H, Yang JL, Yang HM, Zhang LL, Zhang L, et al. A survey of human intestinal protozoa in Yunnan. Chin J Parasitol Parasitic Dis. 1994(S1):99-102. (in Chinese)
142. Fan B, Tao LZ, Xia GH, Liu BY. A report of 2 cases of cryptosporidiosis in infant in Yunnan. Chin J Zoonoses. 1991;7(4):26. (in Chinese)
143. Fan B, He XY, Wang WL, Huang ZM, Su Q. Investigation of *Cryptosporidium* infection in Yuxi County, Yunnan Province. Chin J Parasitol Parasitic Dis. 1994;S1:265. (in Chinese)
144. Teng XJ. Molecular epidemiological investigation of HIV-AIDS complicated with intestinal protozoa infection in Tengchong, Yunnan. Beijing: Chinese Center for Disease Control and Prevention. 2017. (in Chinese)
145. Zhang BX, Yu H, Tao H, Bai ZM, He YQ, Li YL et al. Investigation on intestinal protozoal infection in Yuanyang County, Yunnan Province. Chin J Parasitic Dis Control. 2002;6:22. (in Chinese)
146. Shen LJ, LiW. Investigation of *Cryptosporidium* infection in intravenous drug users in Dali. Chin J Public Health. 2005;11:21-2. (in Chinese)
147. Hou YS, Li JQ, Fei ZD, Chen CH, Tian RH. Investigation of *Cryptosporidium* infection in children in Xi'an area. Chin J Parasitic Dis Control. 1991;3:200. (in Chinese)
148. Hou BX, Hou W. A case of cryptosporidiosis in children. Shaanxi Med J. 1991;20(5):317. (in Chinese)
149. Hou Q, Chen JM, Liu W. Infection and epidemiological characteristics of *Cryptosporidium* in outpatients in a children's hospital in Xi'an. Contemp Med. 2011;17(24):53. (in Chinese)
150. Ling XM, Chen H, Yue W, Mao XR, Song JJ. Investigation of human *Cryptosporidium* infection in special populations. Chin J Parasitic Dis Control. 2001;14(3):1. (in Chinese)
151. Chen H, Mao XR, Ling XM, Song JJ. Epidemiological investigation of *Cryptosporidium* infection in diarrheal patients from three areas of Gansu Province. Chin J Parasitol Parasitic Dis. 2001;19(2):50. (in Chinese)
152. Ling XM, Chen H. Clinical observation of cryptosporidiosis with intestinal bacterial co–infection. J First Mil Meal Univ. 2005;7:919–20. (in Chinese)
153. Wang YN, Dong TT, Wang AX, Dang W, Lu ZL, Zhang S, et al. Epidemiological survey of *Cryptosporidium* and *Entamoeba* *histolytica* infection among patients with a chronic condition in Lanzhou. J Pathog Biol. 2013;8(10):934-6. (in Chinese)
154. Zhao SY. Investigation on the occurrence of cryptosporidiosis in Kang County. Gansu Sci Technol. 1996;12(1):25. (in Chinese)
155. Zhu Y, Zhang YQ. A case report of cryptosporidiosis. 2009;39(10):7. Qinghai Med J. (in Chinese)
156. Li JM, ChenCM. Discussion on the test results of common pathogenic microorganisms for acute diarrhea in children of different genders and ages. World Latest Med Inf. 2019;19(51):166,169. (in Chinese)
157. Zhang B, Zhang YZ, Zuo XJ, Wang X. Identification of *Cryptosporidium* Species from Fecal Samples of One Hospital in Xinjiang. Prog Vet Med. 2017;38(8):53-6. (in Chinese)
158. Tursun M. Detection and analysis of pathogenic microorganisms in stool samples of HIV-infected patients with chronic diarrhea. J Med Inf. 2016;29(31):289-90. (in Chinese)
159. Wang QJ, Zhang J. Investigation of *Cryptosporidium* infection in 1124 children in Tarim region, Xinjiang. Xinjiang Med J. 1995;3:187-8. (in Chinese)
160. Tsaihong JC, Tang RB, Wu KK, Wu TC, Chung CH. Pediatric cryptosporidiosis: a report of 2 cases. Taiwan Yi Xue Hui Za Zhi. 1988;87(9):914-8. (in Chinese)
161. Hung CC, Tsaihong JC, Lee YT, Deng HY, Hsiao WH, Chang SY, et al. Prevalence of intestinal infection due to *Cryptosporidium* species among Taiwanese patients with human immunodeficiency virus infection. J Formos Med Assoc. 2007;106(1):31-5.
162. Huang SH, Lin YF, Tsai MH, Yang S, Liao ML, Chao SW, et al. Detection of common diarrhea-causing pathogens in Northern Taiwan by multiplex polymerase chain reaction. Medicine (Baltimore). 2018;97(23):e11006.
163. Cheng YJ, Lin WC, Liu CE, Ji DD. First microbiologically confirmed case of cryptosporidiosis due to *Cryptosporidium homin*is gp60-If sub-genotype in a patient with HIV-1 infection in Taiwan. J Formos Med Assoc. 2014;113(4):264-5.
