# Supplementary material for: Cryptosporidiosis threat under climate change in China: prediction and validation of habitat suitability and outbreak risk for human-derived Cryptosporidium based on ecological niche models
Source: Infect Dis Poverty. 2023 Apr 11;12:35. doi: 10.1186/s40249-023-01085-0 (PMC10088348; doi:10.1186/s40249-023-01085-0)
Supplement: Supplementary file 5 — Additional file 5. References for human cases of Cryptosporidium in countries neighboring China. [file 40249_2023_1085_MOESM5_ESM.docx]

**Additional file 4** References for human cases of *Cryptosporidium* in countries neighboring of China

| Regions | Countries | Districts | No. points extracted | Investigation time | References |
| --- | --- | --- | --- | --- | --- |
| East Asia | Japan | Kanagawa Prefecture | 1 | 1994 | ^[1]^ |
|  |  | Saitama Prefecture | 1 | 1996 | ^[2]^ |
|  |  | Osaka Prefecture | 1 | 2004 | ^[3]^ |
|  |  | Nagano Prefecture | 1 | 2006 | ^[4]^ |
|  | Korea | Jeollanam-do | 8 | 1992 | ^[5]^ |
|  |  | Seoul City | 2 |  |  |
|  |  | Seoul City | 1 | 1993 | ^[6]^ |
|  |  | Kangwon-do | 1 | 1993 | ^[7]^ |
|  |  | Jeollanam-do | 13 | 2000 | ^[8]^ |
|  |  | Gangwon-do  Chungcheongbuk-do  Gyeongsangnam-do | 1  1  2 | 2001-2002 | ^[9]^ |
|  |  | Jeollanam-do  Jeollabuk-do | 1  1 | 2004 | ^[10]^ |
|  |  | Gyeongsangbuk-do  Jeollanam-do  Gyeongsangnam-do | 1  2  2 | 2005 | ^[11]^ |
| North Asia | Mongolia | Dornod Aimag  Selenge Aimag | 1  1 | 2003 | ^[12]^ |
|  |  | Ulaanbaatar City  Dundgobi Aimag  Zavkhan Aimag  Selenge Aimag | 1  1  1  1 | 2017 | ^[13]^ |
|  | Russia | Tomsk Oblast | 1 | 2017 | ^[14]^ |
| West Asia | Afghanistan | Kabul City | 1 | 2009-2010 | ^[15]^ |
|  |  | Kandahar City | 1 |  |  |
|  | Iran | Fars Province | 1 | 2002 | ^[16]^ |
|  |  | Tehran Province | 1 | 2005-2007 | ^[17]^ |
|  |  | Tehran Province | 1 | Before 2008 | ^[18]^ |
|  |  | Esfahan Province | 2 | 2009-2010 | ^[19]^ |
|  |  | Khuzestan Province | 2 | 2009-2011 | ^[20]^ |
|  |  | Mazandaran Province | 1 | 2011-2012 | ^[21]^ |
|  |  | Esfahan Province | 1 | 2014-2015 | ^[22]^ |
| South Asia | Nepal | Bagmati Zone | 1 | 1996-1997 | ^[23]^ |
|  |  | Gandaki Zone | 1 | Before 2005 | ^[24]^ |
|  |  | Kosi Zone | 1 | 2007-2008 | ^[25]^ |
|  |  | Bagmati Zone | 1 | 2013 | ^[26]^ |
|  | Sri Lanka | Central Province | 1 | 1992-1993 | ^[27]^ |
|  |  | Central Province | 2 | 2011-2013 | ^[28]^ |
|  | Bangladesh | Dhaka Div | 1 | 1985 | ^[29]^ |
|  |  | Dhaka Div | 2 | 2009-2013 | ^[30]^ |
|  |  | Dhaka Div | 1 | 2015-2017 | ^[31]^ |
|  | Pakistan | Punjab Province | 1 | 1996 | ^[32]^ |
|  |  | Sindh Province | 1 | 2008-2010 | ^[33]^ |
|  |  | Baltistan Province | 1 | 2014-2015 | ^[34]^ |
|  |  | Khyber-Pakhtunkhwa Province | 11 | Before 2019 | ^[35]^ |
|  | India | Orissa State | 1 | 1985 | ^[36]^ |
|  |  | West Bengal State | 1 | 1985-1987 | ^[37]^ |
|  |  | Kerala State | 1 | 1987 | ^[38]^ |
|  |  | Uttar Pradesh State | 1 | 1994-1996 | ^[39]^ |
|  |  | Karnataka State | 1 | Before 1995 | ^[40]^ |
|  |  | Tamil Nadu State | 1 | Before 1998 | ^[41]^ |
|  |  | New Delhi City | 1 | 1999-2000 | ^[42]^ |
|  |  | Telangana State | 1 | 2003-2006 | ^[43]^ |
|  |  | Tamil Nadu State | 1 | 2005-2008 | ^[44]^ |
|  |  | Tamil Nadu State | 1 | Before 2007 | ^[45]^ |
|  |  | Chandigarh City | 1 | 2008-2009 | ^[46]^ |
|  |  | Tamil Nadu State | 1 | 2009 | ^[47]^ |
|  |  | Rajasthan State | 1 | 2009 | ^[48]^ |
|  |  | Tamil Nadu State | 3 | Berore 2011 | ^[49]^ |
|  |  | Assam State | 3 | 2014-2016 | ^[50]^ |
| Southeast Asia | Indonesia | East Java Province | 1 | 1992-1993 | ^[51]^ |
|  |  | Jakarta City | 1 | 2004-2007 | ^[52]^ |
|  |  | Maluku Province | 3 | 2015 | ^[53]^ |
|  |  | Sumatera Utara Province | 1 | Before 2019 | ^[54]^ |
|  |  | West Nusa Tenggara Province | 2 | 2019 | ^[55]^ |
|  | Singapore | Singapore City | 1 | 2013-2014 | ^[56]^ |
|  | Cambodia | Phnom Penh City | 1 | 2001 | ^[57]^ |
|  |  | Siem Reap | 1 | Before 2016 | ^[58]^ |
|  | Myanmar | Yangon City | 1 | 1990-1991 | ^[59]^ |
|  |  | Shan State | 1 | 2018 | ^[60]^ |
|  | Laos | Vientiane City | 1 | 2009-2010 | ^[61]^ |
|  |  | Savannakhet Province | 1 |  |  |
|  | Vietnam | Hanam Province | 2 | 2008-2009 | ^[62]^ |
|  |  | Nam Dinh Province | 1 | 2014-2017 | ^[63]^ |
|  | Philippine | Metro Manila | 1 | 1983-1984 | ^[64]^ |
|  |  | Luzon Island | 1 | 2004-2005 | ^[65]^ |
|  |  | Visayas Islands | 1 |  |  |
|  |  | Mindanao Island | 1 |  |  |
|  |  | Northern Samar Province | 2 | 2015 | ^[66]^ |
|  |  | Luzon Island | 1 | 2017 | ^[67]^ |
|  | Malaysia | Kelantan State | 1 | 1996-1998 | ^[68]^ |
|  |  | Penang State | 1 | 1998 | ^[69]^ |
|  |  | Selangor State | 3 | 2004 | ^[70]^ |
|  |  | Selangor State | 1 | 2010-2012 | ^[71]^ |
|  |  | Selangor State | 2 | 2010-2012 | ^[72]^ |
|  |  | Pahang State | 1 |  |  |
|  |  | Ngeri Sembilan State | 1 |  |  |
|  |  | Pahang state | 1 | 2012 | ^[73]^ |
|  |  | Pahang state | 1 | 2017-2018 | ^[74]^ |
|  | Thailand | Loei Changwat | 1 | 1985 | ^[75]^ |
|  |  | Bangkok City | 1 | 1986 | ^[76]^ |
|  |  | Songkla Changwat | 1 | 1995-1996 | ^[77]^ |
|  |  | Kanchanaburi Changwat | 1 | 2001-2002 | ^[78]^ |
|  |  | Bangkok City | 1 | 2009-2010 | ^[79]^ |
|  |  | Ratchaburi Changwat | 1 | 2018-2019 | ^[80]^ |

**References**

1. Kuroki T, Watanabe Y, Asai Y, Yamai S, Endo T, Uni S, et al. An outbreak of waterborne Cryptosporidiosis in Kanagawa, Japan. Kansenshogaku Zasshi. 1996;70(2):132-40.
2. Yamamoto N, Urabe K, Takaoka M, Nakazawa K, Gotoh A, Haga M, et al. Outbreak of cryptosporidiosis after contamination of the public water supply in Saitama Prefecture, Japan, in 1996. Kansenshogaku Zasshi. 2000;74(6):518-26.
3. Takagi M , Toriumi H , Endo T , et al. An Outbreak of Cryptosporidiosis Associated with Swimming Pools. Kansenshogaku Zasshi. 2008;82(1):14-19.
4. Yoshida H, Matsuo M, Miyoshi T, Uchino K, Nakaguchi H, Fukumoto T, et al. An outbreak of cryptosporidiosis suspected to be related to contaminated food, October 2006, Sakai City, Japan. Jpn J Infect Dis. 2007;60(6):405-7.
5. Chai JY, Lee SH, Guk SM, Lee SH. An epidemiological survey of *Cryptosporidium parvum* infection in randomly selected inhabitants of Seoul and Chollanam-do. Korean J Parasitol. 1996;34(2):113-9.
6. Cho MH, Kim AK, Im K. Detection of *Cryptosporidium* oocysts from out-patients of the Severance Hospital, Korea. Korean J Parasitol. 1993;31(3):193-9.
7. Seo M, Huh S, Chai JY, Yu JR. An epidemiological survey on *Cryptosporidium parvum* infection of inhabitants in Chorwon-gun, Kangwon-do. Korean J Parasitol. 2001;39(2):201-3.
8. Park JH, Kim HJ, Guk SM, Shin EH, Kim JL, Rim HJ, et al. A survey of cryptosporidiosis among 2,541 residents of 25 coastal islands in Jeollanam-Do (Province), Republic of Korea. Korean J Parasitol. 2006;44(4):367-72.
9. Yu JR, Lee JK, Seo M, Kim SI, Sohn WM, Huh S, et al. Prevalence of cryptosporidiosis among the villagers and domestic animals in several rural areas of Korea. Korean J Parasitol. 2004;42(1):1-6.
10. Cheun HI, Choi TK, Chung GT, Cho SH, Lee YH, Kimata I, et al. Genotypic characterization of *cryptosporidium* oocysts isolated from healthy people in three different counties of Korea. J Vet Med Sci. 2007;69(10):1099-101.
11. Cheun HI, Cho SH, Lim YY, Lee BC, Kim JY, Ju JW, et al. *Cryptosporidium parvum* in Korea: prevalence in individuals residing in three major river valleys and genetic characteristics of the isolates. J Vet Med Sci. 2010;72(2):167-72.
12. Huh S, Yu JR, Kim JI, Gotov C, Janchiv R, Seo JS. Intestinal protozoan infections and echinococcosis in the inhabitants of Dornod and Selenge, Mongolia (2003). Korean J Parasitol. 2006;44(2):171-4.
13. Barnes AN, Davaasuren A, Baasandavga U, Lantos PM, Gonchigoo B, Gray GC. Zoonotic enteric parasites in Mongolian people, animals, and the environment: Using One Health to address shared pathogens. PLoS Negl Trop Dis. 2021;15(7):e0009543.
14. Starikova EG, Schubina NI, Voronkova OV, et al. Prevalence and Clinical Laboratory Features of Cryptosporidiosis in Children under 5 Years of Age: A Cross-Sectional Study of Hospital Cases of Acute Intestinal Infection. Current Pediatrics Issues. 2018;17(4):316-21.
15. Elyan D, Wasfy M, El Mohammady H, Hassan K, Monestersky J, Noormal B, Oyofo B. Non-bacterial etiologies of diarrheal diseases in Afghanistan. Trans R Soc Trop Med Hyg. 2014;108(8):461-5.
16. Mirzaei M. Prevalence of Cryptosporidium sp. infection in diarrheic and non-diarrheic humans in Iran. Korean J Parasitol. 2007;45(2):133-7.
17. Taghipour N, Nazemalhosseini-Mojarad E, Haghighi A, Rostami-Nejad M, Romani S, Keshavarz A, et al. Molecular epidemiology of cryptosporidiosis in Iranian children, tehran, iran. Iran J Parasitol. 2011;6(4):41-5.
18. Pirestani M, Sadraei J, Dalimi Asl A, Zavvar M, Vaeznia H. Molecular characterization of *Cryptosporidium* isolates from human and bovine using 18s rRNA gene in Shahriar county of Tehran, Iran. Parasitol Res. 2008;103(2):467-72.
19. Izadi M, Jonaidi-Jafari N, Saburi A, Eyni H, Rezaiemanesh MR, Ranjbar R. Cryptosporidiosis in Iranian Farm Workers and Their Household Members: A Hypothesis about Possible Zoonotic Transmission. J Trop Med. 2014;2014:405875.
20. Heidarnegadi S, Mohebali M, Maraghi Sh, Babaei Z, Farnia Sh, Bairami A, et al. *Cryptosporidium spp*. Infection in human and domestic animals. Iran J Parasitol. 2012;7(1):53-8.
21. Sharif M, Daryani A, Kia E, Rezaei F, Nasiri M, Nasrolahei M. Prevalence of intestinal parasites among food handlers of Sari, Northern Iran. Rev Inst Med Trop Sao Paulo. 2015;57(2):139-44.
22. Mohaghegh MA, Hejazi SH, Ghomashlooyan M, Kalani H, Mirzaei F, Azami M. Prevalence and clinical features of *Cryptosporidium* infection in hemodialysis patients. Gastroenterol Hepatol Bed Bench. 2017;10(2):137-42.
23. Ono K, Rai SK, Chikahira M, Fujimoto T, Shibata H, Wada Y, et al. Seasonal distribution of enteropathogens detected from diarrheal stool and water samples collected in Kathmandu, Nepal. Southeast Asian J Trop Med Public Health. 2001;32(3):520-6.
24. Easow JM, Mukhopadhyay C, Wilson G, Guha S, Jalan BY, Shivananda PG. Emerging opportunistic protozoa and intestinal pathogenic protozoal infestation profile in children of western Nepal. Nepal Med Coll J. 2005;7(2):134-7.
25. Amatya R, Poudyal N, Gurung R, Khanal B. Prevalence of *Cryptosporidium* species in paediatric patients in Eastern Nepal. Trop Doct. 2011;41(1):36-7.
26. Bhattachan B, Sherchand JB, Tandukar S, Dhoubhadel BG, Gauchan L, Rai G. Detection of *Cryptosporidium parvum* and *Cyclospora cayetanensis* infections among people living in a slum area in Kathmandu valley, Nepal. BMC Res Notes. 2017;10(1):464.
27. de Silva NR, de Silva HJ, Jayapani VP. Intestinal parasitoses in the Kandy area, Sri Lanka. Southeast Asian J Trop Med Public Health. 1994;25(3):469-73.
28. Sirisena UM, Iddawela WM, Noordeen F, Wickramasinghe S. Prevalence and identification of *Cryptosporidium* species in paediatric patients with diarrhoea. Ceylon Med J. 2014;59(3):75-8.
29. Rahman M, Shahid NS, Rahman H, Sack DA, Rahman N, Hossain S. Cryptosporidiosis: a cause of diarrhea in Bangladesh. Am J Trop Med Hyg. 1990;42(2):127-30.
30. Ehsan AM, Geurden T, Casaert S, Parvin SM, Islam TM, Ahmed UM, et al. Assessment of zoonotic transmission of *Giardia* and *Cryptosporidium* between cattle and humans in rural villages in Bangladesh. PLoS One. 2015;10(2):e0118239.
31. Korpe PS, Gilchrist C, Burkey C, Taniuchi M, Ahmed E, Madan V, et al. Case-Control Study of *Cryptosporidium* Transmission in Bangladeshi Households. Clin Infect Dis. 2019;68(7):1073-79.
32. Iqbal J, Munir MA, Khan MA. *Cryptosporidium* infection in young children with diarrhea in Rawalpindi, Pakistan. Am J Trop Med Hyg. 1999;60(5):868-70.
33. Yakoob J, Abbas Z, Beg MA, Naz S, Khan R, Islam M, et al. Prevalences of *Giardia lamblia* and *Cryptosporidium parvum* infection in adults presenting with chronic diarrhoea. Ann Trop Med Parasitol. 2010 Sep;104(6):505-10.
34. Khushdil A, Murtaza F, Chattha MN. Cryptosporidiosis Among Children Of District Skardu, Pakistan. J Ayub Med Coll Abbottabad. 2016;28(3):575-577.
35. Khan A, Shams S, Khan S, Khan MI, Khan S, Ali A. Evaluation of prevalence and risk factors associated with *Cryptosporidium* infection in rural population of district Buner, Pakistan. PLoS One. 2019;14(1):e0209188.
36. Subramanyam VR, Broadhead RL, Pal BB, Pati JB, Mohanty G. Cryptosporidiosis in children of eastern India. Ann Trop Paediatr. 1989;9(2):122-5.
37. Pal S, Bhattacharya SK, Das P, Chaudhuri P, Dutta P, De SP, et al. Occurrence and significance of *Cryptosporidium* infection in Calcutta. Trans R Soc Trop Med Hyg. 1989;83(4):520-1.
38. Reinthaler FF, Mascher F, Sixl W, Enayat U, Marth E. Cryptosporidiosis in children in Idukki District in southern India. J Diarrhoeal Dis Res. 1989;7(3-4):89-91.
39. Nath G, Choudhury A, Shukla BN, Singh TB, Reddy DCS. Significance of *Cryptosporidium* in acute diarrhoea in North-Eastern India. J Med Microbiol. 1999;48(6):523-6.
40. Shetty M, Brown TA, Kotian M, Shivananda PG. Viral diarrhoea in a rural coastal region of Karnataka India. J Trop Pediatr. 1995;41(5):301-3.
41. Kang G, Mathew MS, Rajan DP, Daniel JD, Mathan MM, Mathan VI, et al. Prevalence of intestinal parasites in rural Southern Indians. Trop Med Int Health. 1998;3(1):70-5.
42. Kaur R, Rawat D, Kakkar M, Uppal B, Sharma VK. Intestinal parasites in children with diarrhea in Delhi, India. Southeast Asian J Trop Med Public Health. 2002;33(4):725-9.
43. Nagamani K, Pavuluri PR, Gyaneshwari M, Prasanthi K, Rao MI, Saxena NK. Molecular characterisation of *Cryptosporidium*: an emerging parasite. Indian J Med Microbiol. 2007;25(2):133-6.
44. Ajjampur SS, Liakath FB, Kannan A, Rajendran P, Sarkar R, Moses PD, et al. Multisite study of cryptosporidiosis in children with diarrhea in India. J Clin Microbiol. 2010;48(6):2075-81.
45. Ramakrishnan K, Shenbagarathai R, Uma A, Kavitha K, Rajendran R, Thirumalaikolundusubramanian P. Prevalence of intestinal parasitic infestation in HIV/AIDS patients with diarrhea in Madurai City, South India. Jpn J Infect Dis. 2007;60(4):209-10.
46. Saigal K, Sharma A, Sehgal R, Sharma P, Malla N, Khurana S. Intestinal microsporidiosis in India: a two year study. Parasitol Int. 2013;62(1):53-6.
47. Chopra RD, Dworkin MS. Descriptive epidemiology of enteric disease in Chennai, India. Epidemiol Infect. 2013;141(5):953-7.
48. Vyas N, Sood S, Sharma B, Kumar M. The Prevalence of Intestinal Parasitic Infestation and the Related Profile of the CD4 (+) Counts in HIV/AIDS People with Diarrhoea in Jaipur City. J Clin Diagn Res. 2013;7(3):454-6.
49. Ajjampur SS, Sarkar R, Allison G, Banda K, Kane A, Muliyil J, et al. Serum IgG response to *Cryptosporidium* immunodominant antigen gp15 and polymorphic antigen gp40 in children with cryptosporidiosis in South India. Clin Vaccine Immunol. 2011;18(4):633-9.
50. Hussain G, Roychoudhury S, Singha B, Paul J. Incidence of *Cryptosporidium andersoni* in diarrheal patients from southern Assam, India: a molecular approach. Eur J Clin Microbiol Infect Dis. 2017;36(6):1023-32.
51. Katsumata T, Hosea D, Wasito EB, Kohno S, Hara K, Soeparto P, et al. Cryptosporidiosis in Indonesia: a hospital-based study and a community-based survey. Am J Trop Med Hyg. 1998;59(4):628-32.
52. Kurniawan A, Karyadi T, Dwintasari SW, Sari IP, Yunihastuti E, Djauzi S, et al. Intestinal parasitic infections in HIV/AIDS patients presenting with diarrhoea in Jakarta, Indonesia. Trans R Soc Trop Med Hyg. 2009;103(9):892-8.
53. Maria DGS, Irene MR, Fransiska M, Rizqiani AK, Yayuk Hartriyanti E, Elsa Herdiana Murhandarwati E. Prevalence of intestinal protozoan infections and association with hygiene knowledge among primary schoolchildren in Salahutu and Leihitu districts, Central Maluku regency, Indonesia. Trop Biomed. 20161;33(3):428-36.
54. Darlan DM, Rozi MF, Andriyani Y, Yulfi H, Saragih RH, Nerdy N. *Cryptosporidium Sp.* Findings and Its Symptomatology among Immunocompromised Patients. Open Access Maced J Med Sci. 2019;7(10):1567-71.
55. Resnhaleksmana E, Wijayanti MA, Artama WT. A potential zoonotic parasite: cryptosporidium parvum transmission in rats, pigs and humans in west lombok, indonesia. Afr J Infect Dis. 2021;15(2):44-51.
56. Chau ML, Hartantyo SH, Yap M, Kang JS, Aung KT, Gutiérrez RA, et al. Diarrheagenic pathogens in adults attending a hospital in Singapore. BMC Infect Dis. 2016;16:32.
57. Chhin S, Harwell JI, Bell JD, Rozycki G, Ellman T, Barnett JM, et al. Etiology of chronic diarrhea in antiretroviral-naive patients with HIV infection admitted to Norodom Sihanouk Hospital, Phnom Penh, Cambodia. Clin Infect Dis;43(7):925-32.
58. Moore CE, Elwin K, Phot N, Seng C, Mao S, Suy K, et al. Molecular Characterization of *Cryptosporidium* Species and *Giardia* duodenalis from Symptomatic Cambodian Children. PLoS Negl Trop Dis. 2016;10(7):e0004822.
59. Aye T, Moe K, Nyein MM, Swe T. Cryptosporidiosis in Myanmar infants with acute diarrhea. Southeast Asian J Trop Med Public Health. 1994;25(4):654-6.
60. Wu Y, Gong B, Liu X, Jiang Y, Cao J, Yao L, et al. Identification of Uncommon *Cryptosporidium viatorum* (a Novel Subtype XVcA2G1c) and *Cryptosporidium andersoni* as Well as Common *Giardia duodenalis* Assemblages A and B in Humans in Myanmar. Front Cell Infect Microbiol. 2020;10:614053.
61. Paboriboune P, Phoumindr N, Borel E, Sourinphoumy K, Phaxayaseng S, Luangkhot E, et al. Intestinal parasitic infections in HIV-infected patients, Lao People's Democratic Republic. PLoS One. 2014;9(3):e91452.
62. Pham-Duc P, Nguyen-Viet H, Hattendorf J, Zinsstag J, Phung-Dac C, Zurbrügg C, et al. *Ascaris lumbricoides* and *Trichuris trichiura* infections associated with wastewater and human excreta use in agriculture in Vietnam. Parasitol Int. 2013;62(2):172-80.
63. Iwashita H, Takemura T, Tokizawa A, Sugamoto T, Thiem VD, Nguyen TH, et al. Molecular epidemiology of *Cryptosporidium spp.* in an agricultural area of northern Vietnam: A community survey. Parasitol Int. 2021;83:102341.
64. Cross JH, Alcantara A, Alquiza L, Zaraspe G, Ranoa C. Cryptosporidiosis in Philippine children. Southeast Asian J Trop Med Public Health. 1985;16(2):257-60.
65. Natividad FF, Buerano CC, Lago CB, Mapua CA, de Guzman BB, Seraspe EB, et al. Prevalence rates of Giardia and Cryptosporidium among diarrheic patients in the Philippines. Southeast Asian J Trop Med Public Health. 2008;39(6):991-9.
66. Weerakoon KG, Gordon CA, Williams GM, Cai P, Gobert GN, Olveda RM, et al. Co-parasitism of intestinal protozoa and *Schistosoma japonicum* in a rural community in the Philippines. Infect Dis Poverty. 2018;7(1):121.
67. Labana RV, Dungca JZ, Nissapatorn V. Community-based surveillance of *Cryptosporidium* in the indigenous community of Boliwong, Philippines: from April to December 2017. Epidemiol Health. 2018;40:e2018047.
68. Menon BS, Abdullah S, Mahamud F, Morgan UM, Malik AS, Choo KE, et al. Low prevalence of *Cryptosporidium parvum* in hospitalized children in Kota Bharu, Malaysia. Southeast Asian J Trop Med Public Health. 2001;32(2):319-22.
69. Ludin CM, Afifi SA, Hasenan N, Maimunah A, Anuar AK. Cryptosporidiosis among children with acute gastroenteritis in the pediatric ward in the General Hospital, Penang. Southeast Asian J Trop Med Public Health. 1991;22(2):200-2.
70. Al-Mekhlafi HM, Mahdy MA, 'Azlin MY, Fatmah MS, Norhayati M. Childhood *Cryptosporidium* infection among aboriginal communities in Peninsular Malaysia. Ann Trop Med Parasitol. 2011;105(2):135-43.
71. Rossle NF, Latif B, Malik AS, Fadzli FM, Abu NA. Cryptosporidiosis among children with diarrhea admitted to hospital Selayang and hospital Sungai Buloh, Selangor, Malaysia. J Trop Med Parasitol. 2012;35(2):55-62.
72. Lee SC, Ngui R, Tan TK, Muhammad Aidil R, Lim YA. Neglected tropical diseases among two indigenous subtribes in peninsular Malaysia: highlighting differences and co-infection of helminthiasis and sarcocystosis. PLoS One. 2014;9(9):e107980.
73. Al-Delaimy AK, Al-Mekhlafi HM, Nasr NA, Sady H, Atroosh WM, Nashiry M, et al. Epidemiology of intestinal polyparasitism among Orang Asli school children in rural Malaysia. PLoS Negl Trop Dis. 2014;8(8):e3074.
74. Asady A, Ismail S, Marsitah AJ, Pakeer O. Prevalence of *Cryptosporidium spp.* infection among children admitted to Hospital Tengku Ampuan Afzan. Med J Malaysia. 2019 ;74(6):468-71.
75. Taylor DN, Echeverria P, Pitarangsi C, Seriwatana J, Sethabutr O, Bodhidatta L, et al. Application of DNA hybridization techniques in the assessment of diarrheal disease among refugees in Thailand. Am J Epidemiol. 1988;127(1):179-87.
76. Thamlikitkul V, Tepmongkol M, Lamon C, Sripochang S, Rungnapawate W, Suvajeejarun T. Cryptosporidiosis in Siriraj Hospital, Bangkok, Thailand. Southeast Asian J Trop Med Public Health. 1987;18(2):229-32.
77. Uga S, Kunaruk N, Rai SK, Watanabe M. *Cryptosporidium* infection in HIV-seropositive and seronegative populations in southern Thailand. Southeast Asian J Trop Med Public Health. 1998;29(1):100-4.
78. Wongstitwilairoong B, Srijan A, Serichantalergs O, Fukuda CD, McDaniel P, Bodhidatta L, et al. Intestinal parasitic infections among pre-school children in Sangkhlaburi, Thailand. Am J Trop Med Hyg. 2007;76(2):345-50.
79. Supcharassaeng S, Suankratay C. Antibiotic prescription for adults with acute diarrhea at King Chulalongkorn Memorial Hospital, Thailand. J Med Assoc Thai. 2011;94(5):545-50.
80. Sutthikornchai C, Popruk S, Mahittikorn A, Arthan D, Soonthornworasiri N, Paratthakonkun C, et al. Molecular detection of *Cryptosporidium spp.*, *Giardia duodenalis*, and *Enterocytozoon bieneusi* in school children at the Thai-Myanmar border. Parasitol Res. 2021;120(8):2887-95.
