# Supplementary material for: Cryptosporidiosis threat under climate change in China: prediction and validation of habitat suitability and outbreak risk for human-derived Cryptosporidium based on ecological niche models
Source: Infect Dis Poverty. 2023 Apr 11;12:35. doi: 10.1186/s40249-023-01085-0 (PMC10088348; doi:10.1186/s40249-023-01085-0)
Supplement: Supplementary file 8 — Additional file 8. Statistics of cities with high, moderate, and low habitat suitability for human-derived Cryptosporidium in China. [file 40249_2023_1085_MOESM8_ESM.docx]

**Additional file 7** Statistics of cities with high, moderate and low habitat suitability of human-derived *Cryptosporidium* in China

| Provinces | Cities (including municipalities, prefectures, leagues, special administrative regions, counties administrated by province and Taiwan Province) | | |
| --- | --- | --- | --- |
|  | With high habitat suitability | With moderate habitat suitability | With low habitat suitability |
| Beijing |  | Beijing (1) |  |
| Tianjin | Tianjin (1) |  |  |
| Hebei | Tangshan, Qinhuangdao, Handan, Xingtai, Cangzhou, Langfang, Hengshui (7) | Shijiazhuang, Baoding (2) | Zhangjiakou, Chengde (2) |
| Shanxi |  | Yangquan, Jincheng, Linfen, Yuncheng (4) | Taiyuan, Datong, Changzhi, Shuozhou, Xinzhou, Lvliang, Jinzhong (7) |
| Inner Mongolia |  | Tongliao (1) | Hohhot, Baotou, Wuhai, Chifeng, Ordos, Hulun Buir, Ulanqab, Hinggan League, Xilingol League (9) |
| Liaoning | Shenyang, Anshan, Dandong, Jinzhou, Yingkou, Liaoyang, Panjin, Tieling, Huludao (9) | Dalian, Fushun, Benxi, Fuxin, Chaoyang (5) |  |
| Jinlin |  | Changchun, Jilin, Siping, Liaoyuan, Tonghua, Baishan, Songyuan, Baicheng (8) | Yanbian (1) |
| Heilongjiang |  | Harbin, Qiqihar, Daqing, Suihua (4) | Jixi, Hegang, Shuangyashan, Yichun, Jiamusi, Qitaihe, Mudanjiang, Heihe (8) |
| Shanghai |  | Shanghai (1) |  |
| Jiangsu | All cities (13) |  |  |
| Zhejiang | Hangzhou, Jiaxing, Huzhou, Shaoxing, Zhoushan (5) | Ningbo, Wenzhou, Jinhua, Quzhou, Lishui (5) |  |
| Anhui | All Cities (16) |  |  |
| Fujian |  | All Cities (9) |  |
| Jiangxi | Nanchang, Jingdezhen, Jiujiang, Xinyu, Yingtan, Ganzhou, Fuzhou (7) | Pingxiang, Ji’an, Yichun, Shangrao (4) |  |
| Shandong | All Cities (16) |  |  |
| Henan | Zhengzhou, Kaifeng, Pingdingshan, Anyang, Hebi, Xinxiang, Puyang, Xuchang, Luohe, Shangqiu, Zhoukou, Zhumadian, Nanyang, Xinyang (14) | Luoyang, Jiaozuo, Sanmenxia, Jiyuan (4) |  |
| Hubei | Wuhan, Huangshi, Xiangyang, Jingzhou, Yichang, Xiaogan, Jingmen, Ezhou, Huanggang, Xianning, Suizhou (11) | Shiyan, Enshi, Xiantao, Qianjiang, Tianmen, Shennongjia (6) |  |
| Hunan | Yueyang, Changde, Zhangjiajie (3) | Changsha, Zhuzhou, Xiangtan, Hengyang, Shaoyang, Yiyang, Loudi, Chenzhou, Yongzhou, Huaihua, Xiangxi (11) |  |
| Guangdong | Zhaoqing (1) | Guangzhou, Shantou, Foshan, Shaoguan, Heyuan, Meizhou, Huizhou, Dongguan, Zhongshan, Jiangmen, Zhanjiang, Maoming, Qingyuan, Chaozhou, Jieyang, Yunfu (16) | Shenzhen, Zhuhai, Shanwei, Yangjiang (4) |
| Guangxi | Nanning, Liuzhou, Wuzhou, Chongzuo, Laibin, Hezhou, Hechi, Guigang (8) | Guilin, Beihai, Yulin, Baise, Qinzhou (5) | Fangchenggang (1) |
| Hainan |  | Sansha, Danzhou, Wuzhishan, Wenchang, Qionghai, Wanning, Dongfang, Ding’an, Tunchang, Chengmai, Lingao, Baisha, Changjiang, Ledong, Lingshui, Baiting, Qiongzhong (17) | Haikou, Sanya (2) |
| Chongqing |  | Chongqing (1) |  |
| Sichuan | Zigong, Luzhou, Suining, Neijiang, Nanchong, Yinbin, Guang’an, Dazhou, Bazhong, Ziyang (10) | Chengdu, Panzhihua, Deyang, Mianyang, Guangyuan, Leshan, Meishan (7) | Ya’an, Liangshan (2) |
| Guizhou |  | All Cities (9) |  |
| Yunnan |  | Kunming, Qujing, Yuxi, Shaotong, Honghe, Wenshan (6) | Baoshan, Lijiang, Puer, Lincang, Dehong, Nujiang, Diqing, Dali, Chuxiong, Xishuangbanna (10) |
| Shaanxi |  | Xi’an, Baoji, Xianyang, Tongchuan, Weinan, Hanzhong, Ankang, Shangluo (8) | Yan’an, Yulin (2) |
| Gansu |  |  | Tianshui, Pingliang, Qingyang, Longnan (4) |
| Ningxia |  | Shizuishan, Wuzhong, Guyuan, Zhongwei (4) |  |
| Xinjiang |  | Shihezi, Alaer, Tumushuke, Wujiaqu, Beitun (5) | Tulufan, Aksu, Bayingol (3) |
| Hong Kong |  | Hong Kong (1) |  |
| Macao |  | Macao (1) |  |
| Taiwan |  | All cities |  |
